# Early childhood wheezing phenotypes and determinants in a South African birth cohort: longitudinal analysis of the Drakenstein Child Health Study

**DOI:** 10.1016/S2352-4642(22)00304-2

**Published:** 2023-02

**Authors:** Carlyle McCready, Sadia Haider, Francesca Little, Mark P Nicol, Lesley Workman, Diane M Gray, Raquel Granell, Dan J Stein, Adnan Custovic, Heather J Zar

**Affiliations:** a Department of Statistical Sciences, University of Cape Town, Cape Town, South Africa; b Department of Paediatrics and Child Health, University of Cape Town, Cape Town, South Africa; c SA-Medical Research Council Unit on Child and Adolescent Health, University of Cape Town, Cape Town, South Africa; d Department of Psychiatry and Mental Health, University of Cape Town, Cape Town, South Africa; e SA-Medical Research Council Unit on Risk and Resilience, University of Cape Town, Cape Town, South Africa; f National Heart and Lung Institute, Imperial College London, London, UK; g Marshall Centre, School of Biomedical Sciences, University of Western Australia, Perth, WA, Australia; h Medical Research Council Integrative Epidemiology Unit, Department of Population Health Sciences, Bristol Medical School, University of Bristol, Bristol, UK

## Abstract

**Background:**

Developmental trajectories of childhood wheezing in low-income and middle-income countries (LMICs) have not been well described. We aimed to derive longitudinal wheeze phenotypes from birth to 5 years in a South African birth cohort and compare those with phenotypes derived from a UK cohort.

**Methods:**

We used data from the Drakenstein Child Health Study (DCHS), a longitudinal birth cohort study in a peri-urban area outside Cape Town, South Africa. Pregnant women (aged ≥18 years) were enrolled during their second trimester at two public health clinics. We followed up children from birth to 5 years to derive six multidimensional indicators of wheezing (including duration, temporal sequencing, persistence, and recurrence) and applied Partition Around Medoids clustering to derive wheeze phenotypes. We compared phenotypes with a UK cohort (the Avon Longitudinal Study of Parents and Children [ALSPAC]). We investigated associations of phenotypes with early-life exposures, including all-cause lower respiratory tract infection (LRTI) and virus-specific LRTI (respiratory syncytial virus, rhinovirus, adenovirus, influenza, and parainfluenza virus) up to age 5 years. We investigated the association of phenotypes with lung function at 6 weeks and 5 years.

**Findings:**

Between March 5, 2012, and March 31, 2015, we enrolled 1137 mothers and there were 1143 livebirths. Four wheeze phenotypes were identified among 950 children with complete data: never (480 children [50%]), early transient (215 children [23%]), late onset (104 children [11%]), and recurrent (151 children [16%]). Multivariate adjusted analysis indicated that LRTI and respiratory syncytial virus-LRTI, but not other respiratory viruses, were associated with increased risk of recurrent wheeze (odds ratio [OR] 2·79 [95% CI 2·05–3·81] for all LTRIs; OR 2·59 [1·30–5·15] for respiratory syncytial virus-LRTIs). Maternal smoking (1·88 [1·12–3·02]), higher socioeconomic status (2·46 [1·23–4·91]), intimate partner violence (2·01 [1·23-3·29]), and male sex (2·47 [1·50–4·04]) were also associated with recurrent wheeze. LRTI and respiratory syncytial virus-LRTI were also associated with early transient and late onset clusters. Wheezing illness architecture differed between DCHS and ALSPAC; children included in ALSPAC in the early transient cluster wheezed for a longer period before remission and late-onset wheezing started at an older age, and no persistent phenotype was identified in DCHS. At 5 years, airway resistance was higher in children with early or recurrent wheeze compared with children who had never wheezed. Airway resistance increased from 6 weeks to 5 years among children with recurrent wheeze.

**Interpretation:**

Effective strategies to reduce maternal smoking and psychosocial stressors and new preventive interventions for respiratory syncytial virus are urgently needed to optimise child health in LMICs.

**Funding:**

UK Medical Research Council; The Bill & Melinda Gates Foundation; National Institutes of Health Human Heredity and Health in Africa; South African Medical Research Council; Wellcome Trust.

## Introduction

Wheezing is common in early childhood and might be caused by a range of different pathophysiological mechanisms.^1^ Birth cohort studies in high-income countries have described several wheezing phenotypes, with specific and shared risk factors.^2^ The process included hypothesis-generating methodologies aimed at identifying the latent structure of patterns of wheezing onset, remission, and recurrence within large datasets^3^ using a variety of unbiased machine learning and statistical methods.^4^ Data-driven approaches have mostly used latent class models such as the latent class analysis^5^, ^6^, ^7^ to describe the within-individual and between-individual variability over a specified interval. The number of reported latent class analysis phenotypes varied by study.^8^ Furthermore, although latent class analysis phenotypes in different studies are usually designated by the same name, they often have different temporal trajectories, distributions within a population, and associated risk factors.^2^, ^8^

In contrast to high-income countries, little information is available on the patterns of childhood wheezing illness in low-income and middle-income countries (LMICs), despite the high prevalence of wheezing illness and large burden of risk factors and adverse environmental exposures associated with wheezing. This includes the high incidence and severity of early-life lower respiratory tract infections (LRTIs),^9^ with respiratory syncytial virus, which is frequently associated with recurrent wheezing in high-income countries, being a predominant causal pathogen.^10^ Furthermore, high exposure to air pollution, tobacco smoke, psychosocial stressors, or poor living conditions might lead to different patterns of wheezing illness in LMICs compared with high-income countries. Therefore, understanding phenotypes of childhood wheezing illness in LMICs is important to understand the spectrum of disease, identify risk factors, and inform intervention strategies.

**Research in context**

**Evidence before this study**

We searched PubMed for articles published in English from database inception until June 15, 2022, using combinations of the search terms “wheezing phenotypes” and “child” or “birth cohort”. We included studies with longitudinal analysis of wheezing phenotypes from birth and into childhood. We identified 18 birth cohorts in high-income countries and none from low-income and middle-income countries (LMICs), despite the high prevalence of wheezing illness, burden of exposures associated with wheezing, and large childhood populations in LMICs. Four or five phenotypes were identified in most cohorts: never or infrequent wheeze, early transient, late onset, recurrent, and persistent wheeze. Studies reported a strong association between early-life respiratory syncytial virus or rhinovirus-lower respiratory tract infection (LRTI) and subsequent recurrent wheezing, but few did active surveillance for LRTI or tested for other viruses. However, wheeze phenotypes and associated risk factors, particularly early-life LRTI or adverse environmental exposures might differ between LMICs and high-income countries. Few studies measured lung function longitudinally from birth or preceding LRTI, with little data available on the association of wheeze phenotypes and lung function.

**Added value of this study**

To our knowledge, this is the first birth cohort to describe patterns of wheezing, associated risk factors, and impact on lung function in a LMIC, where exposures differ to those in high-income countries in previous studies. The Drakenstein Child Health Study is a unique birth cohort with extensive collection of data on wheeze and respiratory viruses at 14 timepoints in the first 5 years of life with high cohort retention. The study is also the only birth cohort in a LMIC in which comprehensive early-life lung function has been assessed using measures preceding wheezing or LRTI, enabling accurate delineation of the impact of these on lung function. We found that wheezing was common, with four specific wheezing phenotypes identified: never wheeze, early transient, late onset, or recurrent. There were specific differences between the wheeze phenotypes described and those from cohorts in high-income countries, with differences in age of onset of specific phenotypes, different viral exposures (with respiratory syncytial virus most strongly associated with wheezing), and absence of a persistent phenotype in our cohort. We also identified specific early life or antenatal exposures highly prevalent in LMICs that were associated with specific wheezing phenotypes. We also found that recurrent wheezing was associated with lung function impairment at age 5 years.

**Implications of all the available evidence**

Specific patterns of wheezing occur in African children. Early exposures highly prevalent in LMICs, were associated with wheezing phenotypes. Respiratory syncytial virus-LRTI was associated with early, late, and recurrent phenotypes. A recurrent wheezing phenotype was associated with lung function impairment in childhood, independent of baseline lung function. Stronger strategies to reduce or prevent such exposures, including new preventive interventions for respiratory syncytial virus, are urgently needed to optimise child health.

We hypothesised that wheeze phenotypes in early childhood and their covariates in LMICs (in particular early-life LRTI) differ from those in high-income countries, and that the causes of early-life LRTI will affect presentation, development, and recurrence of wheezing. We aimed to derive longitudinal wheeze phenotypes from a South African birth cohort and compare those with phenotypes derived from a UK cohort. We also aimed to investigate the associations of wheezing phenotypes with early-life risk factors including specific viral LRTI and baseline lung function at 6 weeks, and explore the association of different wheeze phenotypes with lung function at 5 years.

## Methods

### Study design and participants

For this analysis, we derived longitudinal wheeze phenotypes from birth to 5 years in the Drakenstein Child Health Study (DCHS), a unique South African birth cohort.^11^, ^12^, ^13^ DCHS is a population-based birth cohort, which has been described previously^12^, ^13^ (appendix p 4). Briefly, pregnant women (aged ≥18 years) were enrolled during their second trimester at two public health clinics in a peri-urban area outside Cape Town, South Africa. All births occurred at the single public hospital, where birth parameters were obtained by study staff.

We compared phenotype structure with the same analysis in the Avon Longitudinal Study of Parents and Children (ALSPAC),^14^ a UK birth cohort study. ALSPAC was used as a comparator cohort since this is one of the largest UK cohorts with the greatest number of data collection timepoints in the first 5 years, and therefore provides the most robust comparator.

The DCHS was approved by the Human Research Ethics Committee of the Faculty of Health Sciences, University of Cape Town, South Africa (HREC 401/2009). Mothers provided written informed consent at enrolment and were re-consented annually.

### Procedures

In the DCHS, validated questionnaires were completed at 14 scheduled visits from birth until 5 years (six visits in the first 12 months, and at 6-monthly intervals thereafter). Spells were defined as consecutive wheezing (appendix pp 5–6). For each child, we derived six wheeze indicators:^15^ 1, age at first recorded episode; 2, age at the last recorded episode; 3, total number of separate records over the observation period; 4, duration of the longest spell based on the number of consecutive records of wheeze; 5, total number of spells; 6, spell type (defined as 0=no wheeze, 1=single spell, 2=intermittent spells). An example of the derivation of variables is shown in the appendix (pp 5–6). We used identical methods to derive wheeze phenotypes in ALSPAC (appendix p 5).^14^, ^16^ Definitions of other variables, including socioeconomic status and maternal and child health measures,^11^, ^12^, ^13^ are summarised in the appendix (pp 7–8).

We used active surveillance to confirm LRTI; all episodes were assessed by trained study staff and defined according to WHO definitions.^11^ Episodes of LRTI or of wheezing that occurred 28 days apart or less were recorded as a single episode, since symptoms from viral LRTI might persist, as previously described.^11^, ^17^ At each LRTI or wheezing episode, a nasopharyngeal swab was obtained for multiplex real-time PCR with FTDResp33 (Fast-track Diagnostics, Esch-sur-Alzet, Luxembourg), which is able to identify up to 33 organisms, including respiratory syncytial virus, rhinovirus, adenovirus, influenza, and parainfluenza virus, which have been previously associated with LRTI in this cohort.^11^, ^17^

To collect lung function data, airway oscillometry was performed at 6 weeks in unsedated infants during quiet sleep and at 5 years in children sitting comfortably with a nose clip in place, as previously described.^17^, ^18^ The intra-breath measurements included in the analysis were respiratory resistance at the end of expiration (R_eE_) and respiratory reactance at the end of expiration (X_eE_), points of zero flow. Measurements were collected in healthy children at least 4 weeks after a LRTI or wheezing episode (appendix pp 4–5).

### Statistical analysis

We carried out clustering among 950 children in DCHS with complete data on wheeze across 14 timepoints. To derive wheeze patterns captured by the multidimensional indicators, we used the Partition Around Medoids (PAM) algorithm coupled with the Wishart distance for mixed data, since this provides the most robust method for derivation of phenotypes (appendix p 5).^15^ The algorithm was run for 2–7 clusters, and the optimal number of clusters was chosen using the average of the silhouette width criterion (appendix p 6). To determine the stability of the optimal number of clusters, we ran multiple iterations of the PAM model while sampling random subsets of varying sample size with decrements of 10% from the full set until 50% were included. To determine the impact of model choice, we did latent class analysis with binary variables for wheeze and the optimal number of clusters was chosen using the Bayesian information criterion (appendix p 15).

Multinomial logistic regression was used to investigate associations of each cluster with early-life or environmental risk factors, including viral LRTIs. Results are reported as odds ratios (ORs) with 95% CIs. Unadjusted and adjusted models were fitted using variables based on evidence from cohorts in high-income countries and associations previously published in the DCHS.^17^ Variable selection was based on a backwards selection procedure, starting with a model that included all clinically relevant variables (appendix p 9). The full set of exposures was included as the minimum sufficient set of confounders. A sample size of 950 had sufficient power for clustering and modelling common exposures (appendix p 6).

Where repeated LRTI episodes occurred, the number of LRTI episodes per child was treated as a count variable, whereas binary indicators were used for the presence or absence of respiratory syncytial virus, rhinovirus, adenovirus, influenza, or parainfluenza virus at least once during follow-up.

The association between lung function and wheezing phenotypes was investigated by assessing the predictive power of 6-week lung function values for phenotype allocation through a multinomial logistic model and comparing lung function at 5 years in the wheezing phenotype groups using linear regression models adjusted for sex and height. We compared 6-week and 5-year lung function by phenotype using cohort derived Z scores.

We exponentiated normal confidence intervals on the log-scale to calculate 95% CIs for incidence rate. Proportions were compared using the χ^2^ test or Fisher's exact test where appropriate. Mood's Median Test was used to compare medians. Analyses were conducted in R (version 3.6.3).

### Role of the funding source

The funders of the study had no role in study design, data collection, data analysis, data interpretation, or writing of the report.

## Results

Between March 5, 2012, and March 31, 2015, 1137 mothers (median age 25·8 years [IQR 22·0–30·8]) were enrolled, and there were 1143 livebirths (four sets of twins, one set of triplets). Cohort retention was high (1015 [88·8%] of 1143 children at 1 year; 994 [87·0%] at 2 years; and 981 [85·8%] at 5 years; appendix p 9). This analysis included 950 (83%) of 1143 children with complete data on wheezing across 14 timepoints to 5 years. Excluded children had similar characteristics to those included in the analysis, with the exception of higher socioeconomic status and lower prevalence of antenatal smoking in the excluded group (appendix p 10). There were 24 child deaths: three were due to LRTIs (appendix p 9).

Characteristics of the study population are described in the appendix (p 10). All women and children included in the cohort were of Black African or mixed ancestry and predominantly of low socioeconomic status; 836 (88%) of 950 children were living in households with a monthly income of less than ZAR5000 (<US$367), of whom 374 (39%) lived in a household earning less than ZAR1000 ($67) per month. A third of mothers smoked antenatally (286 [30%] of 950 mothers), and a similar proportion postnatally (306 [32%] of mothers). 206 (22%) of 950 mothers had HIV; all were on antiretroviral therapy and two (<1%) of 950 children had HIV. Mental health issues were common, with more than a fifth of mothers reporting depression (203 [24%] of 845 mothers), psychological distress (174 [21%] of 846 mothers), or intimate partner violence (292 [34%] of 849 mothers) antenatally. Births were distributed evenly across the seasons, with 150 (16%) of 950 babies born preterm, of whom 101 (67%) were late preterm.

At least one episode of wheezing occurred in 470 (49%) of 950 children; 256 (27%) had one, 112 (12%) had two, and 102 (11%) had three or more episodes. Median age of the first wheezing episode was 8·3 months (IQR 3·3–16·8). The prevalence of current wheeze across 14 timepoints peaked at 13% at 6 months and declined to 2% by 5 years (appendix p 11).

980 LRTI episodes were recorded in 459 children (incidence 0·206 episodes per child-year [95% CI 0·194–0·219**]**), of which, admission to hospital was required for 209 LTRIs (21%; table 1). 170 respiratory syncytial virus episodes occurred in 156 children (0·036 episodes per child-year [0·031–0·042]), 298 rhinovirus episodes occurred in 195 children (0·064 episodes per child-year [0·056–0·071]), 136 adenovirus episodes occurred in 116 children (0·028 episodes per child-year [0·024–0·034]), whereas influenza (0·014 episodes per child-year [0·011–0·017]) and parainfluenza (0·016 episodes per child-year [0·012–0·018]) were less common (table 1). The median age of first respiratory syncytial virus-LRTI was 8·22 months (IQR 3·49–13·79), which was younger than for rhinovirus-LRTI (12·26 months [IQR 5·19–23·13]; p<0·0001), adenovirus-LRTI (12·27 months [8·36–16·73]; p<0·0001), influenza (13·41 months [8·68–18·59]; p<0·0001), and parainfluenza (10·31 months [5·51–19·18]; p=0·72). Among children who were admitted to hospital for treatment, respiratory syncytial virus (0·011 episodes per child-year [95% CI 0·008–0·014]) was more frequent than rhinovirus (0·008 episodes per child-year [0·006–0·011]) or adenovirus (0·005 episodes per child-year [0·003–0·007]), influenza (0·001 episodes per child-year [0·000–0·003]), and parainfluenza (0·002 episodes per child-year [0·001–0·004]). Repeated episodes of LRTI occurred in 236 (25%) of 950 children; 12 (1%) had repeated infection with respiratory syncytial virus, 56 (6%) had repeated infection with rhinovirus, and 15 (2%) had repeated infection with adenovirus. Wheezing at the time of LRTI occurred in 339 (35%) of 980 LRTI episodes and was more common among children with respiratory syncytial virus-LRTI (93 [55%] of 170 children; p<0·0001) and rhinovirus-LRTI (167 [56%] of 298 children; p<0·0001) than adenovirus-LRTI (51 [38%] of 136 children), influenza-LRTI (one [1%] of 67 children), or parainfluenza-LRTI (none).

**Table 1:** Distribution of lower respiratory illness in children in the Drakenstein Child Health Study from birth to age 5 years

	**Children, n**	**Episodes, n**	**Children with repeated episodes, n**	**Episodes per child-year (95% CI)**	**Median age at first episode, months (IQR)**
**LTRI**					
All	459	980	236	0·206 (0·194–0·219)	9·24 (3·95–18·57)
Respiratory syncytial virus	156	170	12	0·036 (0·031–0·042)	8·22 (3·49–13·79)
Rhinovirus	195	298	56	0·064 (0·056–0·071)	12·26 (5·19–23·13)
Adenovirus	116	136	15	0·028 (0·024–0·034)	12·27 (8·36–16·73)
Influenza (types A, B, and C)	61	67	6	0·014 (0·011–0·017)	13·41 (8·68–18·59)
Parainfluenza (types 1, 2, 3, and 4)	70	74	4	0·016 (0·012–0·018)	10·31 (5·51–19·18)
**LTRI requiring hospital admission**					
Any	156	209	33	0·044 (0·038–0·051)	5·16 (1·67–12·85)
Respiratory syncytial virus	50	51	1	0·011 (0·008–0·014)	3·02 (1·66–10·55)
Rhinovirus	31	37	5	0·008 (0·006–0·011)	13·25 (5·19–24·66)
Adenovirus	22	23	1	0·005 (0·003–0·007)	10·68 (8·88–16·29)
Influenza (types A, B, and C)	7	7	0	0·001 (0·000–0·003)	9·39 (5·51–13·39)
Parainfluenza (types 1, 2, 3, and 4)	10	10	0	0·002 (0·001–0·004)	7·98 (3·61–10·43)
**Wheezing at the time of LRTI**					
Any	226	339	69	0·071 (0·064–0·079)	8·18 (4·31–14·19)
Respiratory syncytial virus	89	93	4	0·019 (0·016–0·024)	7·36 (3·02–13·22)
Rhinovirus	127	167	24	0·035 (0·031–0·041)	8·02 (4·75–14·15)
Adenovirus	48	51	1	0·011 (0·008–0·014)	12·00 (8·69–16·70)
Influenza (types A, B, and C)	1	1	0	..	..
Parainfluenza (types 1, 2, 3, and 4)	0	0	0	..	..

LRTI=lower respiratory tract infection.

The optimal solution based on the average of the silhouette width criterion suggested four phenotypes (appendix p 11). After inspection of the trajectories of wheeze, age of onset, and duration of wheeze, the phenotypes were characterised as: 1) never wheeze (480 [50%] of 950 children); 2) early transient wheeze (215 [23%] of 950 children), in which wheeze occurred from age 1·4 months, peaked at 6 months (30%), but remitted after 18 months; 3) late-onset wheeze (104 [11%] of 950 children), with wheeze from 12 months onwards, peaked at 18 months (34%), and declined to 4% by 5 years; and 4) recurrent wheeze (151 [16%] of 950 children), in whom wheeze occurred intermittently throughout (figure A).

**Figure:**
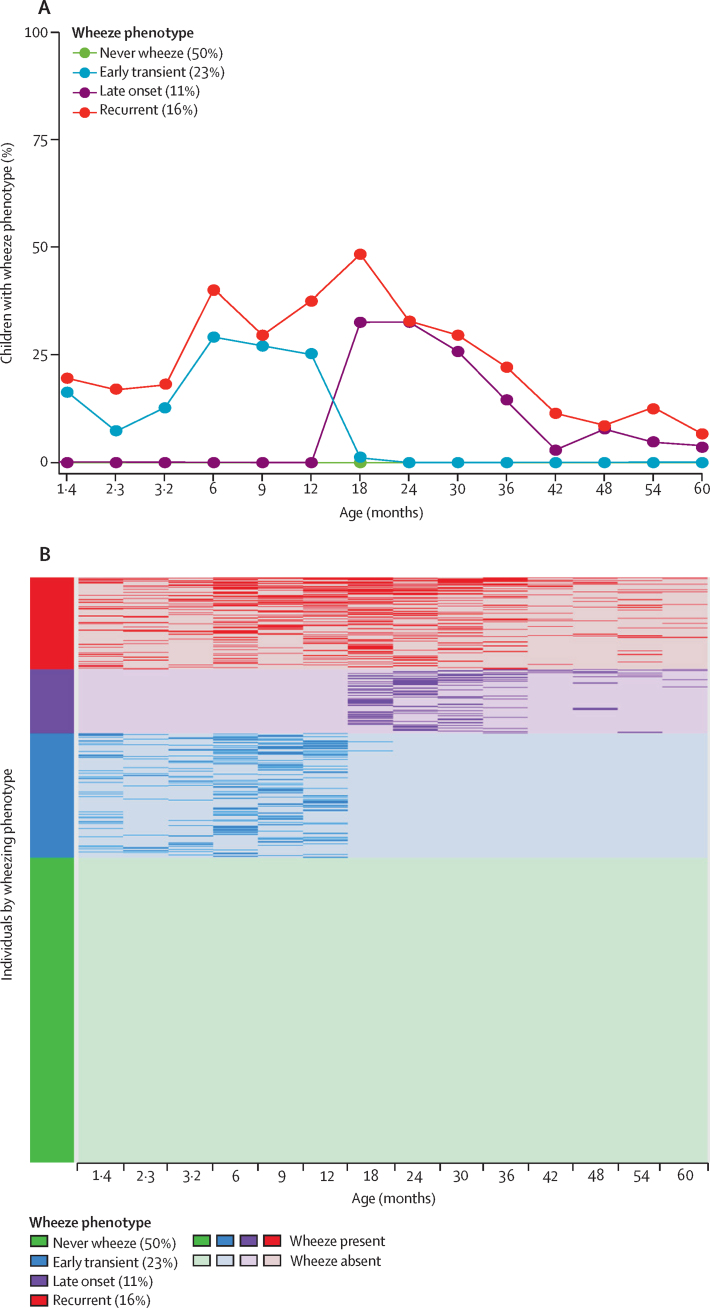
Characteristics of four wheeze phenotypes identified in the Drakenstein Child Health Study (A) Proportion of children with wheezing up to age 5 years for each wheeze phenotype. (B) Intra-individual wheeze patterns, stratified by phenotype; the darker colours of the bars or lines represent the presence of wheeze and lighter colours represent the absence of wheeze.

Intra-individual wheeze patterns were stratified by phenotype (figure B). The distribution of the derived indicators stratified by phenotype and their clinical characteristics are shown in the appendix (pp 12–13). Recurrent wheeze was the only phenotype where children had more than three wheeze episodes, and spells that comprised 3–8 consecutive wheezing events. Early transient and late-onset wheeze were characterised with less frequent, shorter, and a greater proportion of single spells.

The 4-phenotype solution was stable to changes in sample size (appendix p 14), although there was minor variation with regard to timing of wheeze resolution in the early transient phenotype, and start in the late-onset cluster (appendix p 14). For comparison with the optimal spell-based solution, we investigated the structure of the 4-class latent class analysis solution. The phenotypes were highly heterogeneous with regard to intra-individual wheezing patterns within each phenotype and were less interpretable compared with the PAM phenotypes (appendix p 15).

We derived wheeze clusters for 6754 children from ALSPAC who had complete data on wheeze at 6, 16, 30, 42, 57, and 69 months. The overall proportion of children with at least one wheeze episode was similar to that in DCHS (3489 [52%] of 6754 children in ALSPAC *vs* 470 [49%] of 950 children in DCHS). However, at 6 months, approximately twice as many children in ALSPAC had a wheeze episode than in DCHS (1635 [24%] of 6754 children *vs* 126 [13%] of 950 children), and by 5 years, wheeze prevalence declined to 18% (1233 of 6754 children) in ALSPAC compared with 1% (14 of 950 children) in DCHS (appendix p 15). A different architecture of wheezing illness was identified in ALSPAC, with a 5-cluster solution deemed optimal (appendix p 16): never wheeze (3265 [48%] of 6754 children), early transient (1596 [24%]), late onset (551 [8%]), intermittent (783 [12%]), and persistent (559 [8%]; appendix p 17). A cluster unique to ALSPAC (persistent wheeze) was characterised by a prolonged period of wheeze across any timepoints. The other four clusters seemed similar, but children in the early transient wheeze cluster wheezed for a longer period before remission in ALSPAC than DCHS (42 months *vs* 18 months), and late-onset wheezing started later in ALSPAC than DCHS (42 months *vs* 18 months).

The distribution of early-life respiratory syncytial virus-LRTI, rhinovirus-LRTI, adenovirus-LRTI, influenza-LRTI, and parainfluenza-LRTI stratified by wheeze phenotype is shown in table 2. There were marked differences in the prevalence of viral LRTIs across wheeze phenotypes; LRTI was most commonly associated with recurrent wheezing (125 [83%] of 151 children) compared with other phenotypes (124 [26%] of 480 children with never wheeze; 143 [67%] of 215 children with early transient wheeze; 67 [64%] of 104 children with late-onset wheeze). LRTIs requiring hospital admission was also highest among children with recurrent wheezing (60 [40%] of 151 children) compared with other phenotypes (33 [7%] of 480 children with never wheeze; 44 [20%] of 215 children with early transient wheeze; 19 [18%] of 104 with late-onset wheeze). Among hospitalised cases, respiratory syncytial virus was the most common viral-LRTI among children with recurrent wheezing.

**Table 2:** Number of children with respiratory syncytial virus, rhinovirus, adenovirus, influenza, or parainfluenza-LRTI, by wheezing phenotype

	**Never wheeze (n=480)**	**Early transient (n=215)**	**Late onset (n=104)**	**Recurrent (n=151)**	**Total (n=950)**
All-cause LRTI	124 (26%)	143 (67%)	67 (64%)	125 (83%)	459 (48%)
All-cause LRTI requiring hospital admission	33 (7%)	44 (20%)	19 (18%)	60 (40%)	156 (16%)
Respiratory syncytial virus-LRTI	20 (4%)	57 (26%)	22 (15%)	57 (35%)	156 (16%)
Respiratory syncytial virus-LRTI requiring hospital admission	7 (1%)	16 (7%)	3 (3%)	24 (16%)	50 (5%)
Rhinovirus-LRTI	35 (7%)	49 (23%)	32 (31%)	79 (52%)	195 (21%)
Rhinovirus-LRTI requiring hospital admission	1 (<1%)	4 (2%)	9 (9%)	17 (11%)	31 (3%)
Adenovirus-LRTI	19 (4%)	31 (14%)	21 (20%)	45 (30%)	116 (12%)
Adenovirus-LRTI requiring hospital admission	2 (<1%)	6 (3%)	3 (3%)	11 (7%)	22 (2%)
Influenza (type A, B, or C)-LRTI	11 (2%)	20 (9%)	12 (12%)	18 (12%)	61 (6%)
Influenza (type A, B, or C)-LRTI requiring hospital admission	1 (<1%)	2 (1%)	1 (1%)	3 (2%)	7 (1%)
Parainfluenza (type 1, 2, 3, or 4)-LRTI	12 (3%)	17 (8%)	10 (10%)	31 (21%)	70 (7%)
Parainfluenza (type 1, 2, 3, or 4)-LRTI requiring hospital admission	0	1 (<1%)	2 (2%)	7 (5%)	10 (1%)

Children could have more than one type of LRTI; LTRI categories were not mutually exclusive. LRTI=lower respiratory tract infection.

Maternal and infant characteristics, socioeconomic status, all-cause LRTI, and specific viral LRTI were associated with wheezing phenotypes (appendix p 19). Not all strong univariate associations entered the adjusted model after the model building process (appendix p 7). Postnatal and antenatal maternal characteristics were highly correlated, with a stronger association between postnatal exposures and wheeze phenotypes than between antenatal exposures and wheeze phenotypes, which were therefore included in the adjusted model (table 3). The associations in the univariate models between LRTI and wheeze phenotypes persisted in the multivariate model but with smaller effect sizes. LRTI and respiratory syncytial virus-LRTI were associated with all three wheezing phenotypes, whereas other respiratory viruses were not strongly associated with any phenotype. Exclusive breastfeeding was protective, especially for early transient wheeze. In addition to all-cause LRTI and respiratory syncytial virus-LRTI, maternal smoking, intimate partner violence, higher socioeconomic status, and male sex were associated with recurrent wheezing (table 3).

**Table 3:** Adjusted multinomial logistic regression of factors associated with wheezing phenotypes in the Drakenstein Child Health Study

		**Phenotype 2: early transient wheeze**		**Phenotype 3: late-onset wheeze**		**Phenotype 4: recurrent wheeze**	
		OR (95% CI)	p value	OR (95% CI)	p value	OR (95% CI)	p value
**LRTI**							
Number of LRTI episodes		1·69 (1·27–2·30)	0·00053	1·60 (1·13–2·28)	0·0081	2·79 (2·05–3·81)	<0·0001
Hospital admission (*vs* no hospital admission)		1·40 (0·43–1·54)	0·52	0·84 (0·38–1·82)	0·66	1·01 (0·51–2·02)	0·97
Respiratory syncytial virus-LRTI (*vs* respiratory syncytial virus-negative)		3·34 (1·82–6·26)	<0·0001	2·82 (1·32–6·01)	0·0071	2·59 (1·30–5·15)	0·0067
Rhinovirus-LRTI (*vs* rhinovirus-negative)		1·39 (0·74–2·59)	0·31	1·79 (0·87–3·73)	0·12	1·64 (0·83–3·22)	0·15
Adenovirus-LRTI (*vs* adenovirus-negative)		0·87 (0·42–1·77)	0·71	1·56 (0·73–3·43)	0·21	1·11 (0·53–2·30)	0·78
Influenza (type A, B, or C)-LRTI *vs* influenza negative		1·36 (0·46–2·77)	0·78	1·38 (0·51–3·73)	0·52	0·74 (0·27–1·95)	0·54
Parainfluenza (type 1, 2, 3, or 4)-LRTI *vs* parainfluenza negative		1·05 (0·41–2·62)	0·92	1·75 (0·63–4·81)	0·27	1·15 (0·45–2·92)	0·76
**Maternal characteristics**							
Postnatal smoking		1·14 (0·75–1·74)	0·52	1·65 (1·01–2·73)	0·048	1·88 (1·12–3·02)	0·015
Maternal asthma or allergy		2·13 (1·01–4·49)	0·047	2·02 (0·81–4·99)	0·12	2·43 (0·94–6·26)	0·066
Intimate partner violence		1·23 (0·83–1·83)	0·29	1·71 (1·04–2·80)	0·031	2·01 (1·23–3·29)	0·0051
**Infant characteristics**							
Sex (male *vs* female)		1·29 (0·88–1·87)	0·19	1·44 (0·89–2·33)	0·13	2·47 (1·50–4·04)	0·00033
Exclusive breast feeding at 6 weeks		0·65 (0·45–0·97)	0·035	0·82 (0·50–1·32)	0·42	0·72 (0·44–1·16)	0·18
**Socioeconomic status**							
Asset ownership							
	Low-medium *vs* low	1·31 (0·80–2·16)	0·28	2·51 (1·19–5·27)	0·016	1·22 (0·63–2·31)	0·55
	Medium-high *vs* low	0·86 (0·50–1·50)	0·62	2·12 (0·97–4·63)	0·057	0·89 (0·43–1·82)	0·75
	High *vs* low	1·40 (0·79–2·46)	0·24	4·74 (2·20–10·21)	<0·0001	2·46 (1·23–4·91)	0·017

Reference class was the never wheezing phenotype. OR=odds ratio. LRTI=lower respiratory tract infection.

Airway oscillometry was successfully done in 658 (79%) of 834 infants at 6 weeks and 599 (81%) of 737 infants at 5 years (appendix p 9). Compared with children who never wheezed, X_eE_ was lower at 6 weeks in children with recurrent, but not transient or late-onset wheezing (appendix p 19). R_eE_ at 6 weeks was not associated with any phenotype. At 5 years, R_eE_ was higher among children with early transient wheeze (average increase 0·57 hPa s L^–1^ [95% CI 0·08–1·06]) or recurrent wheeze (average increase 0·79 hPa s L^–1^ [95% CI 0·22–1·36]) than children who never wheezed (table 4). Airway resistance increased between 6 weeks and 5 years among children with recurrent wheeze (appendix p 21). Analysis of residuals indicated no deviations from underlying linear model assumptions (appendix p 21).

**Table 4:** Associations of lung function at 5 years and wheeze phenotypes in the Drakenstein Child Health Study

	**R_eE_ (hPa s L^−1^)**		**X_eE_ (hPa s L^−1^)**	
	Regression coefficient (95% CI)	p value	Regression coefficient (95% CI)	p value
Early transient wheeze *vs* never-wheeze	0·57 (0·08 to 1·06)	0·026	−0·09 (−0·36 to 0·18)	0·512
Late-onset wheeze *vs* never-wheeze	0·34 (−0·30 to 0·97)	0·291	−0·18 (−0·44 to 0·16)	0·298
Recurrent wheeze *vs* never-wheeze	0·79 (0·22 to 1·36)	0·005	−0·23 (−0·55 to 0·09)	0·151

Linear regression models were adjusted for sex and height. R_eE_=respiratory resistance at the end of expiration. X_eE_=respiratory reactance at the end of expiration.

## Discussion

To our knowledge, this is the first birth cohort to describe patterns of wheezing, associated risk factors, and impact on lung function in a LMIC from birth to age 5 years. Wheezing was common in this African birth cohort in a poor peri-urban community, with almost half of infants having at least one episode of wheezing in the first 5 years of life. However, wheeze frequency in this cohort was lower than the ALSPAC cohort in the UK and wheezing prevalence declined more rapidly. We identified four wheeze clusters in DCHS: never wheeze (50%), early transient (23%), late onset (11%), and recurrent wheeze (16%), with differences in age of onset and absence of a persistent phenotype compared with those in ALSPAC. Specific early-life exposures, common in LMICs, were associated with recurrent wheezing, including LRTI, respiratory syncytial virus-LRTI, maternal smoking, and intimate partner violence. Respiratory syncytial virus-LRTI was associated with early, late, and recurrent wheeze phenotypes, but LRTI due to other respiratory viruses (rhinovirus, adenovirus, influenza, parainfluenza-virus) occurred at an older age and was not associated with any wheeze phenotype. Objective measurements of lung function from age 6 weeks to 5 years, preceding wheezing or LRTI, allowed for delineation of the impact of these on lung function, with increased airway resistance at 5 years of age in the recurrent phenotype.

We clustered the multidimensional variables using PAM rather than latent class analysis on binary variables since sensitivity analyses showed that PAM produced more homogeneous and clinically interpretable phenotypes than latent class analysis. Furthermore, the application of PAM to binary wheezing outcomes were not clinically interpretable (appendix p 22). Therefore, it is likely that the derived indicators were, primarily, the precursor for deriving more internally homogeneous phenotypes. The wheezing phenotypes identified in this LMIC setting might seem similar to those from data-driven analyses in birth cohorts in high-income countries. However, most cohorts from high-income countries identified five wheeze phenotypes.^5^, ^15^, ^19^ Although the phenotypes seemed similar, these differed in DCHS and ALSPAC. For example, wheezing in the early phenotype resolved earlier in DCHS than ALSPAC (18 months *vs* 42 months), late wheezing started earlier in DCHS (12 months *vs* 42 months), and no persistent wheeze phenotype was identified in DCHS. These differences indicate quicker resolution of early wheezing, but younger age for development of late wheezing in DCHS than in ALSPAC. Such differences might be partly due to the high burden of respiratory syncytial virus-LRTI as a key precipitant of wheezing phenotypes in this setting, with the highest prevalence in infants. Global estimates report a major proportion of respiratory syncytial virus-LRTI in infancy, with the burden heavily concentrated in LMICs.^10^

Early-life LRTI was one of the strongest associates of all three wheezing clusters, with the strongest association with the recurrent phenotype. Respiratory syncytial virus-LRTI was associated with early, late, and recurrent wheezing. We previously reported that early-life respiratory syncytial virus-LRTI was associated with recurrent wheezing at age 2 years in DCHS;^17^ the current study extends these findings, showing the association to persist at age 5 years. Whether respiratory syncytial virus is causal, a marker of genetic or physiological susceptibility to wheezing, or both is unclear. Nevertheless, the potential for new effective preventive interventions for respiratory syncytial virus, such as maternal vaccination or a long-acting monoclonal antibody, raises possibilities for prevention of respiratory syncytial virus-LRTI, with potential impact on the development of recurrent wheezing.^20^ By contrast, no other respiratory viruses were associated with any of the three wheezing phenotypes. In cohorts from high-income countries, early-life rhinovirus infection was associated with subsequent recurrent wheezing or asthma,^21^, ^22^ but we did not find this in DCHS, highlighting another difference between LMIC and high-income country contexts. However, this difference might be due to the absence of genotyping for rhinovirus in DCHS since only rhinovirus-C has been associated with an increased risk for recurrent wheezing.^22^, ^23^ Our results are also consistent with our previous findings that rhinovirus is not strongly associated with LRTI in DCHS.^11^

Tobacco smoke exposure has been strongly associated with early transient wheeze or other wheeze phenotypes in high-income countries.^24^ In the current study, maternal smoking prevalence was high, with self-reported smoking occurring in almost a third of women. Tobacco smoke exposure was an important risk factor for late and recurrent wheeze phenotypes, highlighting the need to strengthen and target smoking cessation programmes to women of childbearing age. Intimate partner violence was also a risk factor for the recurrent phenotype, consistent with findings in low-income families in the USA that maternal perceived stress is associated with childhood wheeze.^25^ This also extends our previous findings from DCHS, in which postnatal intimate partner violence was associated with wheezing or recurrent wheezing from birth to age 2 years,^26^ and with impaired lung function at 1 year.^27^

Of concern, lung function testing showed that both early and recurrent wheezing were associated with increased airways resistance at 5 years, most marked in children with recurrent wheeze. Increased resistance reflects narrowed airways, which are likely to be a consequence of impairment that has developed in early life, possibly due to LRTI, smoke exposure, or other insults. Consistent with this, we have previously shown that LRTI in early childhood in the DCHS cohort resulted in impairment in lung function from birth to age 2 years.^17^, ^28^ Furthermore, we found that airway resistance increased more between infancy and age 5 years in the recurrent wheeze group than other phenotypes, potentially setting these children on a trajectory for impaired respiratory (but also cardiovascular and metabolic)^29^ health into adulthood.

A limitation of our study was the use of caregiver-reported wheezing, which might have resulted in over-reporting or under-reporting of wheeze. However, we have previously shown strong agreement between caregiver-reported wheezing and health worker-ascertained wheeze (κ=0.8) in the DCHS cohort.^17^ Cases of wheezing or LRTI might have been missed; however, we established strong surveillance systems^11^, ^17^ with close follow-up (14 study visits for wheeze ascertainment and six lung function visits), and all hospital admissions occurred at a single public hospital. The follow-up period was up to 5 years; longer-term follow-up would provide insight into persistence of trajectories through adolescence. However, our analyses enabled definition of pre-school wheezing phenotypes over a period of time when the incidence of LRTI and onset of wheezing is highest.^5^, ^6^, ^7^, ^8^, ^9^, ^10^ Although the inclusion of many potential predictors in a full model limits confounding, we cannot exclude possible unmeasured confounding. Excluded participants had higher socioeconomic status, which is associated with a higher prevalence of wheezing,^30^ but lower rates of maternal smoking compared with those included. However, considering the high retention rates in the cohort, inclusion of the vast majority of participants with complete, comprehensive data collection across 14 timepoints, and because relocation was the most common reason for loss to follow-up, it is unlikely that selection bias is a major concern.

We did not include measurements of atopic sensitisation, but asthma or allergic disease were rarely reported or diagnosed either in children or mothers, which might reflect underdiagnosis in LMICs where infectious diseases are a greater priority and where underdiagnosis of asthma, including severe disease, has been well described.^30^ Our findings might not be generalisable to other geographical areas; however, this cohort has many features that are common in other LMICs, including low socioeconomic status, high burden of infectious diseases, high rates of maternal HIV, psychosocial stressors, and exposure to tobacco smoke. These limitations were mitigated by several strengths, including careful longitudinal phenotyping and high retention, meticulous surveillance for LRTI, including delineating cause, and measurements of lung function from 6 weeks of age.

In summary, to our knowledge, this is the first study from a LMIC to show that childhood wheezing is common, with four distinct phenotypes identified. Early exposures highly prevalent in LMICs (maternal smoking, intimate partner violence, LRTI, and respiratory syncytial virus-LRTI) were associated with specific wheezing phenotypes. LRTI was an important risk factor associated with wheezing, with respiratory syncytial virus a key driver of recurrent wheezing. The recurrent wheeze phenotype was associated with lung function impairment at age 5 years, independent of baseline function. Effective strategies to reduce such exposures, including smoking cessation programmes in women of childbearing age, reducing psychosocial stressors, and new preventive interventions for respiratory syncytial virus are urgently needed to optimise child health, especially in LMICs.

## Data sharing

An anonymised, de-identified version of the dataset can be made available on request to allow all results to be reproduced. All requests should be directed to Heather Zar, the Principal Investigator.

## Declaration of interests

HJZ reports grants from the Bill & Melinda Gates Foundation, the NIH H3 Africa, the UK Medical Research council (MRC), Wellcome Trust, and the South African MRC. DG reports grants from the Wellcome Trust. AC reports personal fees from Stallergenes Greer; and personal fees from AstraZeneca, GlaxoSmithKline, and Worg Pharmaceuticals, outside the submitted work. All other authors declare no competing interests.
