# Supplementary material for: Early childhood wheezing phenotypes and determinants in a South African birth cohort: longitudinal analysis of the Drakenstein Child Health Study
Source: Lancet Child Adolesc Health. 2023 Feb;7(2):127–35. doi: 10.1016/S2352-4642(22)00304-2 (PMC9870786; doi:10.1016/S2352-4642(22)00304-2)
Supplement: Supplementary appendix [file mmc1.pdf]

THE LANCET
Child & Adolescent Health

Supplementary appendix

This appendix formed part of the original submission and has been peer reviewed. We post it as supplied by the authors.

Supplement to: McCready C, Haider S, Little F, et al. Early childhood wheezing phenotypes and determinants in a South African birth cohort: longitudinal analysis of the Drakenstein Child Health Study. *Lancet Child Adolesc Health* 2022; published online Nov 23. https://doi.org/10.1016/S2352-4642(22)00304-2.

Supplementary appendix

Early childhood wheezing phenotypes and determinants in a
South African birth cohort: longitudinal analysis of the
Drakenstein Child Health Study

Carlyle McCready, Sadia Haider, Francesca Little, Mark P Nicol, Lesley Workman, Diane M. Gray, Raquel Granell, Dan J Stein, Adnan Custovic, Heather J. Zar

Department of Statistical Sciences (C McCready MSc, Prof F Little PhD), Department of Paediatrics and Child Health (C McCready, L Workman MPH, D M Gray PhD, Prof H J Zar PhD), SA-Medical Research Council Unit on Child and Adolescent Health (C McCready, L Workman, D M Gray, Prof H J Zar), Department of Psychiatry and Mental Health (Prof D J Stein PhD), and SA-Medical Research Council Unit on Risk and Resilience (Prof D J Stein), University of Cape Town, Cape Town, South Africa; National Heart and Lung Institute, Imperial College London, London, UK (S Haider PhD, Prof A Custovic PhD); Medical Research Council Integrative Epidemiology Unit, Department of Population Health Sciences, Bristol Medical School, University of Bristol, Bristol, UK (R Granell PhD); Marshall Centre, School of Biomedical Sciences, University of Western Australia, Perth, WA, Australia (Prof M P Nicol PhD)

Contents

METHODS 4
Drakenstein Child Health Study (DCHS) 4
Definition of variables 4
Current wheeze 4
Early-life risk factors 4
Lower Respiratory Tract Infection (LRTI) 4
Lung function 4
Avon Longitudinal Study of Parents and Children (ALSPAC) 5
Statistical analysis 5
Sample size 6
PAM Clustering 6
Selection of the optimal number of clusters and model stability 6
Association of wheeze phenotypes with early−life risk factors and lung function 7
R packages 7
RESULTS 10

Supplementary Tables

Table S1: Derivation of indicators 5
Table S2: Statistical power 6
Table S3: Definition of variables in the Drakenstein Child Health study 7
Table S4: Comparison of included versus excluded children in DCHS 10
Table S5: Distribution of the derived indicators stratified by phenotype 12
Table S6: Clinical characteristics by phenotype 13
Table S7: BIC values for the LCA model in DCHS 15
Table S8: Unadjusted multinomial logistic regression of the association of early−life factors with wheezing phenotypes 19

Supplementary Figures

Figure S1: Directed acyclic graph (DAG) for exposures in the multinomial logistic regression model 9
Figure S2: Participant flow in the DCHS 10
Figure S3: Point prevalence of current wheeze in children in the DCHS by age 11
Figure S4: Average silhouette width to determine the optimal number of clusters using the PAM algorithm 11
Figure S5 Boxplot of the distribution of the average silhouette index by the number of clusters (over random samples with size reducing in decrements of 10% from 100% to half the original sample size). 14
Figure S6 Profiles of wheeze phenotypes over time – Analysis of class stability with respect to changes in different sample sizes[1] of data 14
Figure S7: Characteristics of 4 wheeze phenotypes identified in the DCHS using LCA 15
Figure S8: Point prevalence of current wheeze in ALSPAC in the first 5 years 15

Figure S9: Plot of average silhouette width in ALSPAC to determine optimal number of clusters using PAM algorithm 15

Figure S10: Characteristics of 5 wheeze phenotypes identified in ALSPAC 17

Figure S11: Airway resistance at ages 6 weeks and 5 years in four wheeze clusters in DCHS 21

Figure S12: Analysis of residuals plots assessing linearity of data, homogeneity of residuals variance, and normality of residuals.. 21

Figure S13: Average silhouette width to determine the optimal number of clusters using the PAM algorithm - application of PAM to binary wheezing outcomes……………………….……..…22

Figure S14: Characteristics of 4 wheeze phenotypes identified in DCHS - application of PAM to binary wheezing outcomes……………………………………………………………….......22

METHODS

Drakenstein Child Health Study (DCHS)

Pregnant women were recruited at antenatal clinics at 2 public health facilities in a peri-urban area in South Africa between 5 March 2012 to 31 March 2015 during their second trimester of pregnancy.[1] Inclusion criteria were 18 years or older, 20-28 week gestation, and resident in the area. All births occurred at the single public hospital, where birth parameters were obtained by study staff. The study was approved by the Faculty of Health Sciences Human Research Ethics Committee, University of Cape Town and Western Cape Provincial Research committee.

Mother-child pairs were followed from birth with study visits synchronised with immunization visits (diphtheria, tetanus, acellular pertussis, *H. influenzae* b and inactivated polio vaccine at 6, 10, 14 weeks and 18 months, measles vaccine at 9 and 18 months and 13-valent PCV at 6 weeks, 14 weeks and 9 months). Additional study visits were done 2-weekly in the first year in an intensive subset, and thereafter 6-monthly through 5 years in all.

Follow-up and cohort retention were optimized through community workers, a dedicated study phone line available to all participants at all times and intensive face to face follow-up of the cohort. Disenrollment followed at least 3 unsuccessful attempts (phone calls and home visits) by study staff to locate participants.

Definition of variables

Current wheeze

Wheezing was assessed using ISAAC questionnaires or were diagnosed on auscultation by trained study staff at a study visit or during an intercurrent illness[3]. Current wheeze was defined as a positive response to the question "Has your child had wheezing or whistling in the chest in the last 12 months?" at each follow-up.

Early-life risk factors

Data on risk factors for wheezing from the antenatal period through 5 years were collected, including sociodemographic factors, nutrition, maternal physical and mental health, home environment, birth factors and breast feeding[1], Table S2. Maternal mental health measures included measurements of depression, psychological distress, and intimate partner violence (IPV) antenatally and postnatally[2]. Smoking was assessed by maternal self-report antenatally and postnatally. Socioeconomic status (SES) was assessed through a validated measure comprising 4 components: household income, asset ownership, household size and maternal education [1], Table S2.

Lower Respiratory Tract Infection (LRTI)

Active surveillance was used to confirm LRTI[3,4]; all episodes were assessed by trained study staff and defined by WHO case definitions as:

(1) episode of LRTI (cough or difficulty breathing and increased respiratory rate or lower chest wall in-drawing in a child aged >2 months); or

(2) severe LRTI (child aged <2 months with increased respiratory rate or lower chest wall in-drawing, or any general danger sign in a child of any age).

At each LRTI or wheezing episode, a nasopharyngeal swab (FLOQSwabsTM, Copan Diagnostics, CA) was obtained. Nucleic acid was extracted using mechanical lysis on a Tissuelyzer LT (Qiagen, Germany) followed by extraction with the QIAsymphony® Virus/Bacteria mini kit (Qiagen, Germany). Quantitative multiplex real-time PCR (qPCR) was done using FTDResp33 (Fast-track Diagnostics, Luxembourg), identifying up to 33 organisms including respiratory syncytial virus (RSV), rhinovirus (RV) and adenovirus (AV).[4]

Lung function

Airway oscillometry was performed at 6 weeks in unsedated infants during quiet sleep and at 5 years in children sitting comfortably, nose clip in place, the cheeks firmly supported and breathing through a mouthpiece and filter, in accordance with published consensus guidelines. Oscillometry measures were obtained using custom made equipment as described[5-6] (INCIRCLE wavetube system, University of Szeged, Hungary). The oscillometry system included a loudspeaker, wave-tube and pneumotachograph. Two different oscillometry measurements were collected. First, the conventional measurement of respiratory system impedance (Zrs) spectra, using a pseudorandom signal and second, a single Hz tracking signal was used to follow the intra-breath changes in Zrs. For infants the speaker generated a pseudo-random signal at 8-48 Hz or a single sinusoid of 16Hz, as previously published.[6] For the children a 6-32 Hz signal or a single sinusoid of 10 Hz was delivered at the mouth. Measurements consisted of a minimum of three acceptable measurements (conventional oscillometry) and one

epoch of single frequency (intra-breath), which included a minimum of five regular breaths, i.e. without any vocal cord noise, apnoea, irregular breathing pattern, glottic closure, leak or sighs.

The intra-breath measurements included in analysis were R_{rs} at end expiration (R_{eE}) and X_{rs} at end expiration (X_{eE}), points of zero flow. These measures may be more sensitive to detect associations with respiratory disease as they are less influenced by the changes within the breathing cycle.[6] Measurements were done with a maximum of five 30 second (infants) or 16 second (children) epochs of composite signals to yield a minimum of 3 acceptable measurements and a 60 second recording at single frequency (16Hz at 6 weeks and 10Hz for children >3 years) to obtained a minimum of 5 acceptable breaths for intra-breath measures as described.[6]

Avon Longitudinal Study of Parents and Children (ALSPAC)

ALSPAC is a birth cohort study established in 1991 in Avon, UK.[7] Pregnant women with expected dates of delivery 1st April 1991 to 31st December 1992 were invited to take part in the study. The initial number of pregnancies enrolled is 14,541. Of these initial pregnancies, there were 14,062 live births and 13,988 children who were alive at 1 year of age.

Ethical approval for the study was obtained from the ALSPAC Ethics and Law Committee and the Local Research Ethics Committees. Informed consent for the use of data collected via questionnaires and clinics was obtained from participants following the recommendations of the ALSPAC Ethics and Law Committee at the time. The study website contains details of available data through a fully searchable data dictionary: http://www.bristol.ac.uk/alspac/researchers/our-data/.

Validated questionnaires were completed on multiple occasions from infancy to adolescence.[8] For this analysis, we used data collected at follow-ups at 6, 8, 30, 42, 57, and 69 months). Current wheeze was defined as a positive response to the question "Has your child had wheezing or whistling in the chest in the last 12 months?".

Statistical analysis

Table S1: Derivation of indicators

Table S1 shows how the 6 multi-dimensional variables were derived from the raw binary wheeze variables at 6 time-points. A spell is defined as beginning when wheeze is first observed and ending when non-wheeze is subsequently observed. In the example below, spell lengths can range from one to six consecutive time-points, and individuals can experience multiple spells over the observation period. The variable "Spell type" is a categorical variable with 3 possible outcomes: No wheeze (a child who was never observed to have wheezed over the observation period); Single spell (a child with one spell of wheeze; this can be as short as a single record or as long as the entire observation period if the child wheezed consecutively at all time-points); Intermittent (a child with multiple spells of wheeze; spells are interspersed with observations of no wheeze).

	Wheeze presence/absence						***Derived indicators***					
**ID**	**TP1**	**TP2**	**TP3**	**TP4**	**TP5**	**TP6**	**Length of longest spell**	**Number of separate spells**	**Number of wheeze observations**	**Spell type**	**Time of wheeze onset**	**Time-point of last wheeze observation**
1	1	1	1	1	1	1	6	1	6	Single	1	6
2	1	0	1	1	1	1	4	2	5	Intermittent	1	6
3	0	1	1	1	1	1	5	1	5	Single	2	6
4	1	0	1	0	1	1	2	3	4	Intermittent	1	6

5	0	0	1	1	1	1	4	1	4	Single	3	6
6	1	0	0	0	1	1	2	2	3	Intermittent	1	6
7	0	0	0	0	0	0	0	0	0	No wheeze	3	4

Sample size

Given the exploratory nature of cluster analysis, there are no clear guidelines on the sample size requirements or the relationship between the number of clusters and the number of clustering variables used. A simulation study found that increasing the sample size from 10 to 30 times the number of clustering variables substantially improves the clustering solution; there are decreasing marginal returns thereafter, however, noticeable improvements are evident up to a sample size of 100 times the number of variables[8]. Accordingly, with 6 variables, the sample size of 950 participants is sufficient for clustering. We tested the stability of the cluster solution by simulating changes in sample size, Figure S5, and found the optimal solution to be stable. Table S2 shows the power of associations between risk factors and wheezing phenotypes based on the prevalence of the risk factors, the lowest probability of any specific wheeze phenotype in the unexposed and the average effect size observed in univariate analyses and shows that the sample size of 950 is well powered to detect associations for prevalent exposures.

Table S2: Statistical power

Exposure	P(Exposure)	P(Wheeze Phenotype|Unexposed)	OR	Power
LRTI	0.48	0.05	2.5	95%
RSV	0.16	0.1	3	99%
RV	0.2	0.1	2.0	87%

PAM Clustering

PAM is a clustering algorithm that partitions the dataset into a predefined number of clusters and has the advantage of being robust to noise and the presence of outliers[9]. The algorithm selects k-medoid initially and then swaps the medoid object with non-medoid thereby improving the quality of clusters.

The algorithm is based on an iterative procedure that starts with the selection of a representative object for each group. This is called a medoid and represents the most centrally located object within the cluster. Once the medoids have been selected, the remaining objects are assigned to each cluster by minimizing their distance from medoids. The quality of the partition is then measured by the average dissimilarity between an object and the medoid of its cluster. The algorithm selects k-medoids and then swaps each medoid object with a non-medoid thereby improving the quality of clusters.

Selection of the optimal number of clusters and model stability

With regards to the selection of the optimal number of clusters, the average silhouette width (ASW) has been suggested for finding the number of clusters with PAM[10]. It is a simple measurement of cluster quality that does not rely on statistical model assumptions, and is widely used and trusted for comparing the quality of clustering produced by various clustering methods over different numbers of clusters. Furthermore, the silhouette width achieved robust results in the extensive simulation study of Arbelaitz et al.[11] To test the sensitivity of the optimal number of clusters to different indices, we also checked Pearson's Gamma[12], Dunn[12], and Calinski & Harabasz[13] indices. As the results were consistent across all indices, and for brevity, we report the ASW in the manuscript.

Whilst statistical judgements informed the optimal number of classes, we did not rely solely on the ASW, but also visualisations of the internal structure to check for within-class homogeneity, intra-class separation, and guidance from literature on previously derived wheeze clusters. Importantly, clinical judgement was an integral part of the phenotype derivation process.

Model stability was assessed through comparing the optimal solution using random subsets of samples of varying sizes. The data were first permuted by ID to ensure that the data was ordered randomly, and for each sample size, the PAM algorithm was run for 10 iterations. We then compared the mean ASW for each sample size over 10 iterations.

Association of wheeze phenotypes with early−life risk factors and lung function

We started with a full model containing all possible predictors for our wheezing outcome as indicated in the DAG and included 1) all-cause LRTI and 2) viral-specific LRTI (RSV, RV, AV, Influenza, and Parainfluenza) in our model to investigate the associations between LRTI and wheezing phenotypes.

For additional possible predictors that may be confounding for LRTI variables and for each other, we conducted backwards selection. From the full model, weaker associations were deleted one variable at a time and the impact of the deletion on the coefficients of and other variables was assessed. Weaker associations were those with the larger p-values.

Successive models were compared after the removal of a weaker predictor using BIC and AIC values. The model building process continues until we no longer saw an improvement in model fit while still retaining strong associations. Deletions that would have resulted in a change in regression coefficients for the LRTI and other variables were retained in the model.

The stability of the variables included in the adjusted model and the estimates of the associations were confirmed through cross-validation by refitting the model on randomly selected subsamples. Multicollinearity was assessed using a variance inflation factor (VIF) (ensuring that values did not exceed 10).

Linearity of associations were confirmed by comparing models with the continuous predictors to models with a categorical version of the predictor (LR p-value = 0·51 for Ree; and LR p-value = 0·23), and further compared to splines models for B-spline basis matrix for a polynomial spline with 3 (LR p-value = 0·046 for Ree; and LR p-value = 0·29) and 5 (LR p-value = 0·087 for Ree; and LR p-value = 0·078) degrees of freedom.

Model diagnostics were used to assess the assumptions underlying our linear regression models. The residual (versus fitted) plot showed no fitted pattern. Homogeneity of variance of the residuals was assessed through a plotting the square root of the standardised residuals against the fitted values; a random scatter was observed. QQ plots showed no clear deviations from normality. Analysis of residuals indicated no deviations from underlying linear model assumptions, Figure S12.

R packages

PAM models were fit in R version 3.6.3 (2020-02-29) by using the cluster library (version 2.1.0). Multinomial logistic regression models were fit in R version 3.6.3 (2020-02-29) by using the nnet library (version 7.3.12). Linear models were fit in R version 3.6.3 (2020-02-29) by using the stats library (version 3.6.3). LCA models were fit in R version 3.6.3 (2020-02-29) by using the poLCA library (version 1.4.1)

Table S3: Definition of variables in the Drakenstein Child Health study

Name	Measurement used
**Maternal characteristics**	
Smoking	Self-reported smoking was assessed using the Alcohol, Smoking and Substance Involvement Screening Test (ASSIST) during the past three months antenatally and postnatally.[14]
Maternal asthma or allergy	Self-reported maternal asthma or allergy was assessed by direct interview antenatally.
Depression	The Edinburgh Postnatal Depression Scale (EPDS) was used to measure maternal depression antenatally and postnatally. 10 Questions were scored 0-3 and totalled. A cut-off value of 13 was used to separate the participants into above- or below-threshold groups[1,15]
Psychological distress	The Self-Reported Questionnaire 20-item (SRQ20)[1,15] was used to measure maternal psychological distress antenatally and postnatally. Each item was measured 0-1; a cut-off value of 8 was used to distinguish an above- or below-threshold group.[1,15]
Intimate partner violence (IPV)	IPV Questionnaire adapted from the WHO multi-country study was used to assess maternal physical, emotional, or sexual violence exposure antenatally and postnatally.[1,15]
**Child characteristics**	
Preterm; late preterm	Gestational age at birth < 37 weeks; late preterm gestational age 34 to <37 weeks

Lower Respiratory Tract Infection (LRTI)	World Health Organization (WHO) criteria were used to define LRTI. Episodes of LRTI ≤28 days apart were regarded as a single episode.
RSV-LRTI; RV-LRTI; AV-LRTI; Influenza (A or B or C)-LRTI; Parainfluenza (1,2, 3 or 4)-LRTI	A LRTI episode with a positive PCR result for RSV, RV, AV, Influenza (A or B or C), or Parainfluenza (1,2, 3 or 4) LRTI on a nasopharyngeal swab
Current wheeze	Data on wheezing was obtained using validated questionnaires based on the ISAAC methodology at 14 scheduled visits from birth to 5-years or detected by auscultation by trained study staff. In addition, questionnaires were done at any unscheduled intercurrent illness episode.
Exclusive breast feeding (at 6-weeks)	Maternal reported breastfeeding only at 6-weeks
Antibiotic exposure	Exposure to antibiotics from birth to 5 years as recorded by dispensing records or prescriptions.
**Socio economic status (SES)**	
Household Income	Average household income per month at maternal enrolment. Categories are: Less than R1 000 ($67), R1 000 ($67) to R5 000 ($336), More than R5 000 ($336).
Education	Highest maternal education level obtained. The levels are primary; some secondary; completed secondary education; any tertiary education.
Asset ownership	Asset ownership is a summed score of 13 different questions including: access to electricity, tap or running water, domestic servant, flush toilet inside, built-in kitchen sink, an electric stove or hotplate, working telephone, at least one motor car or truck, motorcycle or scooter, a bicycle, shop at supermarkets, use any financial services, account at a retail store. The levels are: Low, Low-Medium, Medium-High, High.
Household size	The distribution across quartiles of household size (members). The levels are: Small [1-4], Small-Medium [4-5], Medium-Large [5-7], Large [7-18].

Figure S1: Directed acyclic graph (DAG) for evaluation of exposures in the multinomial logistic regression model

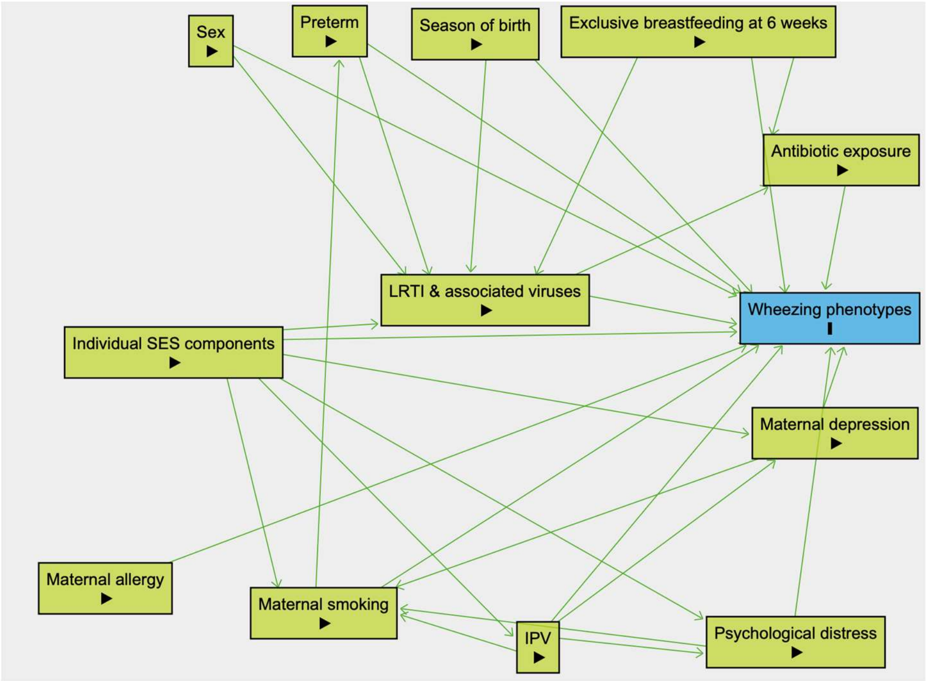

Figure S2: Participant flow in the DCHS

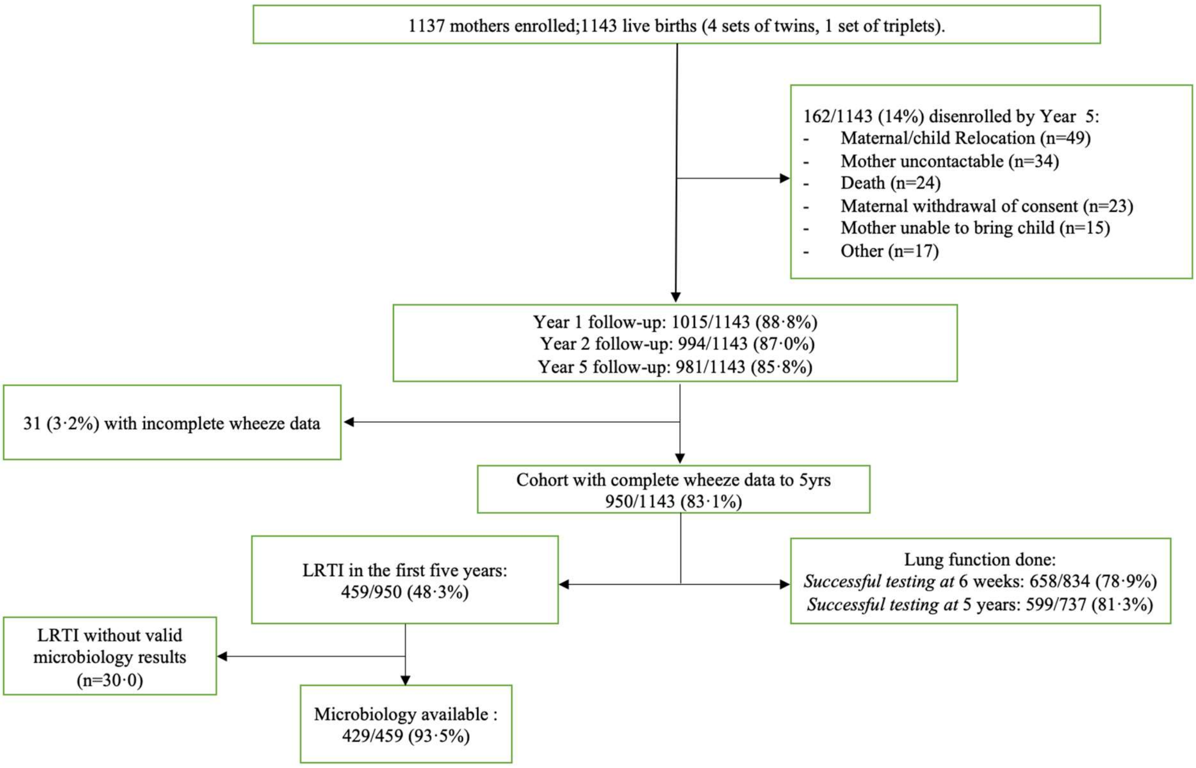

LRTI = Lower Respiratory Tract Infection
**Cause of death: LRTI (n=3), sudden infant death syndrome (n=3), gastroenteritis (n=2), prematurity (n=2), apnoea (n=2), liver failure (n=1), congenital syphilis (n=1), pulmonary atresia (n=1), unknown (n=7)*

RESULTS

Table S4: Comparison of included versus excluded children in DCHS

	Included (N=950)	Excluded or Lost to Follow Up at 5 years of age (N=193)	P-value
**Maternal characteristics**			
Median age [IQR] at enrolment (years)	25·8 [22·0-30·8]	24·4 [21·7-30·2]	p = 0·061
Antenatal smoking	286/950 (30·1%)	38/193 (19·7%)	p < 0·0001
Maternal allergy	55/879 (6·3%)	10/182 (5·5%)	p = 0·82
Antenatal depression	203/845 (24·0%)	34/149 (22·8%)	p = 0·97
Antenatal psychological distress	174/846 (20·6%)	27/145 (18·6%)	p = 0·67
Antenatal IPV	292/849 (34·4%)	42/150 (28·0%)	p = 0·15
Mode of delivery (Caesarean)	188/950 (19·8%)	41/193 (21·2%)	p = 0·72
**Infant characteristics**			
Sex (male)	481/950 (50·6%)	105/193 (54·4%)	p = 0·38
Pre-term (<37 weeks)	150/950 (15·8%)	41/193 (21·2%)	p = 0·081
Late preterm (34 to <37 weeks)	101/950 (10·6%)	22/193 (11·4%)	p = 0·85
HIV exposed uninfected	206/950 (21·7%)	42/193 (21·7%)	p = 0·99
Exclusive Breast Feeding at 6 weeks	452/950 (47·6%)	52/175 (29·7%)	p < 0·0001
Season of birth			
Summer	241/950 (25·4%)	46/193 (23·8%)	p = 0·71
Autumn	239/950 (25·2%)	55/193 (28·5%)	p = 0·37
Winter	256/950 (26·9%)	50/193 (25·9%)	p = 0·83
Spring	214/950 (22·5%)	42/193 (21·8%)	p = 0·89
Median weight-for-age z-score at birth [IQR]	-0·57 [-1·33; 0·09]	-0·55 [-1·21; -0·04]	p = 0·87
**Socio economic status (SES)**			
Income			
< ZAR1 000 ($67)	374/950 (39·4%)	57/193 (29·5%)	p = 0.013
ZAR1 000 -5 000 ($67-336)	462/950 (48·6%)	95/193 (49·2%)	p = 0.94
> ZAR5 000 ($336)	114/950 (12·0%)	41/193 (21·2%)	P = 0.00095
Asset ownership			
Low	242/950 (25·5%)	55/193 (28·5%)	p = 0.43
Low-Medium	296/950 (31·2%)	47/193 (24·3%)	p = 0.073
Medium-High	221/950 (23·3%)	40/193 (20·7%)	p = 0.51
High	191/950 (20·1%)	51/193 (26·4%)	p = 0.063
Household size			
Small [1-4)	302/948 (31·8%)	79/193 (40·9%)	p = 0.018
Small-Medium [4-5)	182/948 (19·2%)	32/193 (16·6%)	p = 0.45
Medium-Large [5-7)	259/948 (27·3%)	48/193 (24·9%)	p = 0.54
Large [7-18]	205/948 (21·6%)	34/193 (17·6%)	p = 0.25
Education			
Primary	70/950 (7·4%)	16/193 (8·3%)	p = 0.76
Some secondary	523/950 (55·1%)	86/193 (44·6%)	p = 0.0097
Completed secondary	303/950 (31·9%)	72/193 (37·3%)	p = 0.17
Any tertiary	54/950 (5·7%)	19/193 (9·8%)	p = 0.046
Lung function (oscillometry) at 6-weeks			
R_{eE} ($hPa \cdot s \cdot L^{-1}$)	42·8 [37·1; 50·9]	40·8 [33·5; 50·4]	p = 0·68
X_{eE} ($hPa \cdot s \cdot L^{-1}$)	-6·7 [-11·9; -2·9]	-6·4 [-10·8; -2·2]	p = 0·51

LRTI = Lower Respiratory Tract Infection; IPV = intimate partner violence; R_{eE} = Respiratory resistance at the end of expiration; X_{eE} = Respiratory reactance at the end of expiration

Figure S3: Point prevalence of current wheeze in children in the DCHS by age

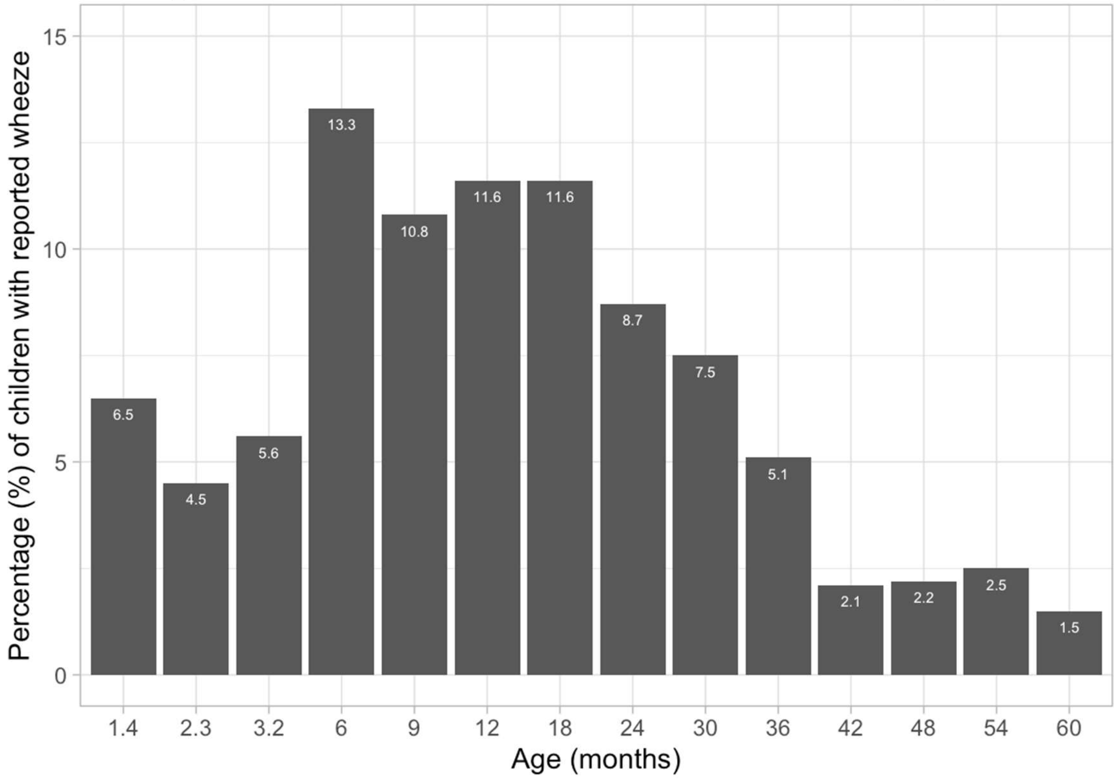

n=62 (1.4 months); n=43 (2.3 months); n=53 (3.2 months); n=126 (6 months); n=103 (9 months); n=110 (12 months); n=110 (18 months); n=83 (24 months); n=71 (30 months); n=48 (36 months); n=20 (42 months); n=21 (48 months); n=24 (54 months); n=14 (60 months)

Figure S4: Average silhouette width to determine the optimal number of clusters using the PAM algorithm

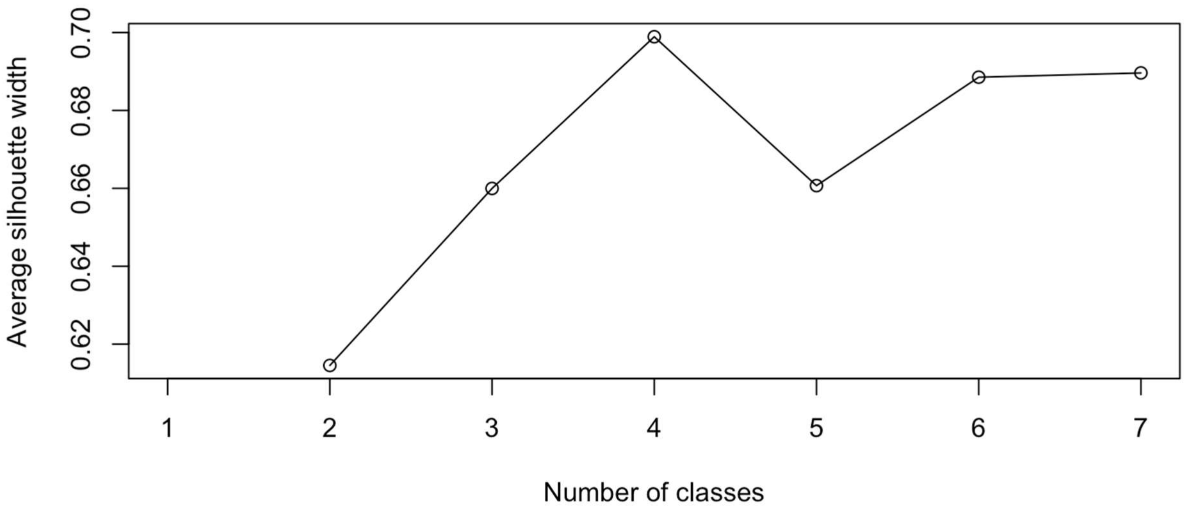

Table S5: Distribution of the derived indicators stratified by phenotype

a) Total number of separate wheeze episodes

	Never wheeze		Transient early		Late onset		Recurrent	
**0**	480	(100·0%)	0	(0·0%)	0	(0·0%)	0	(0·0%)
**1**	0	(0·0%)	176	(81·9%)	80	(76·9%)	0	(0·0%)
**2**	0	(0·0%)	39	(18·1%)	22	(21·1%)	51	(33·8%)
**3**	0	(0·0%)	0	(0·0%)	2	(1·9%)	51	(33·8%)
**4**	0	(0·0%)	0	(0·0%)	0	(0·0%)	25	(16·6%)
**5**	0	(0·0%)	0	(0·0%)	0	(0·0%)	10	(6·6%)
**6**	0	(0·0%)	0	(0·0%)	0	(0·0%)	4	(2·6%)
**7**	0	(0·0%)	0	(0·0%)	0	(0·0%)	7	(4·6%)
**8**	0	(0·0%)	0	(0·0%)	0	(0·0%)	1	(0·7%)
**9**	0	(0·0%)	0	(0·0%)	0	(0·0%)	2	(1·3%)
**Total**	**480**	**(100%)**	**215**	**(100%)**	**104**	**(100%)**	**151**	**(100%)**

b) Total number of wheeze spells

	Never wheeze		Transient early		Late onset		Recurrent	
**0**	480	(100·0%)	0	(0·0%)	0	(0·0%)	0	(0·0%)
**1**	0	(0·0%)	199	(92·6%)	94	(90·4%)	11	(7·3%)
**2**	0	(0·0%)	16	(7·4%)	9	(8·6%)	100	(66·2%)
**3**	0	(0·0%)	0	(0·0%)	1	(1·0%)	35	(23·2%)
**4**	0	(0·0%)	0	(0·0%)	0	(0·0%)	4	(2·6%)
**5**	0	(0·0%)	0	(0·0%)	0	(0·0%)	1	(0·7%)
**Total**	**480**	**(100%)**	**215**	**(100%)**	**104**	**(100%)**	**151**	**(100%)**

c) Longest spell based on the number of consecutive records of wheeze

	Never wheeze		Transient early		Late onset		Recurrent	
**0**	480	(100·0%)	0	(0·0%)	0	(0·0%)	0	(0·0%)
**1**	0	(0·0%)	192	(89·3%)	89	(85·6%)	68	(45·0%)
**2**	0	(0·0%)	23	(10·7%)	15	(14·4%)	46	(30·6%)
**3**	0	(0·0%)	0	(0·0%)	0	(0·0%)	21	(13·9%)
**4**	0	(0·0%)	0	(0·0%)	0	(0·0%)	7	(4·6%)
**5**	0	(0·0%)	0	(0·0%)	0	(0·0%)	4	(2·6%)
**6**	0	(0·0%)	0	(0·0%)	0	(0·0%)	4	(2·6%)
**8**	0	(0·0%)	0	(0·0%)	0	(0·0%)	1	(0·7%)
**Total**	**480**	**(100%)**	**215**	**(100%)**	**104**	**(100%)**	**151**	**(100%)**

d) Spell type

	Never wheeze		Transient early		Late onset		Recurrent	
**Intermittent[1]**	0	(0·0%)	16	(7·4%)	10	(9·6%)	141	(93·4%)
**Single**	0	(0·0%)	199	(92·6%)	94	(90·4%)	10	(6·6%)
**No wheeze**	480	(100·0%)	0	(0·0%)	0	(0·0%)	0	(0·0%)
**Total**	**480**	**(100%)**	**215**	**(100%)**	**104**	**(100%)**	**151**	**(100%)**

[1]intermittent defined as at least 2 non-consecutive spells of wheeze of any leng

Table S6: Clinical characteristics by phenotype

Infant Characteristics	Never wheeze (n=480)	Early transient (n=215)	Late onset (n=104)	Recurrent (n=151)
Sex (male)	224 (46·7%)	101 (47·0%)	55 (52·9%)	101 (66·9%)
Pre-term (<37 weeks)	74 (15·4%)	27 (12·6%)	18 (17·3%)	31 (20·5%)
HIV (exposed)	109 (22·7%)	52 (24·2%)	19 (18·3%)	26 (17·2%)
Exclusive Breast Feeding at 6 weeks	249 (51·9%)	86 (40·0%)	47 (45·2%)	70 (46·3%)
Season of Birth				
Autumn	97 (20·2%)	67 (31·1%)	27 (25·9%)	48 (31·8%)
Spring	117 (24·4%)	38 (17·7%)	29 (27·9%)	30 (19·8%)
Summer	129 (26·9%)	49 (22·8%)	27 (25·9%)	36 (23·8%)
Winter	152 (31·7%)	48 (22·3%)	21 (20·2%)	35 (23·2%)
Antibiotic exposure	125 (26·0%)	120 (55·8%)	68 (65·4%)	122 (80·7%)
Median weight-for-age z-score at birth [IQR]	-0·45 [-1·24; 0·18]	-0·67 [-1·35; 0·11]	-0·77 [-1·45; -0·11]	-0·67 [-1·36; -0·01]
**Maternal Characteristics**				
Mode of delivery (Caesarean)	95 (19·8%)	45 (20·9%)	24 (23·1%)	24 (15·9%)
Antenatal Smoking	122 (25·4%)	63 (29·3%)	40 (38·5%)	61 (40·4%)
Postnatal Smoking	139 (28·9%)	63 (29·3%)	41 (39·4%)	63 (41·7%)
Maternal Allergy	22 (4·6%)	14 (6·5%)	8 (7·7%)	11 (7·3%)
Antenatal Depression	108 (22·5%)	35 (16·3%)	21 (20·2%)	39 (25·8%)
Postnatal Depression	115 (23·9%)	47 (21·9%)	23 (22·1%)	44 (29·1%)
Antenatal Psychological Distress	79 (16·5%)	36 (16·7%)	17 (16·3%)	42 (27·8%)
Postnatal Psychological Distress	57 (11·9%)	26 (12·1%)	14 (13·5%)	31 (20·5%)
Antenatal IPV	152 (31·7%)	52 (24·2%)	30 (28·8%)	58 (38·4%)
Postnatal IPV	156 (32·5%)	76 (35·3%)	45 (42·3%)	78 (51·6%)

IPV = intimate partner violence; HIV = human immunodeficiency virus

Figure S5: Boxplot of the distribution of the average silhouette index by the number of clusters (over random samples with size reducing in decrements of 10% from 100% to half the original sample size). The plot shows that the optimal solution across the 6 iterations was 4 classes. Outliers are defined as < Q1 – 1.5*IQR or > Q3 + 1.5*IQR

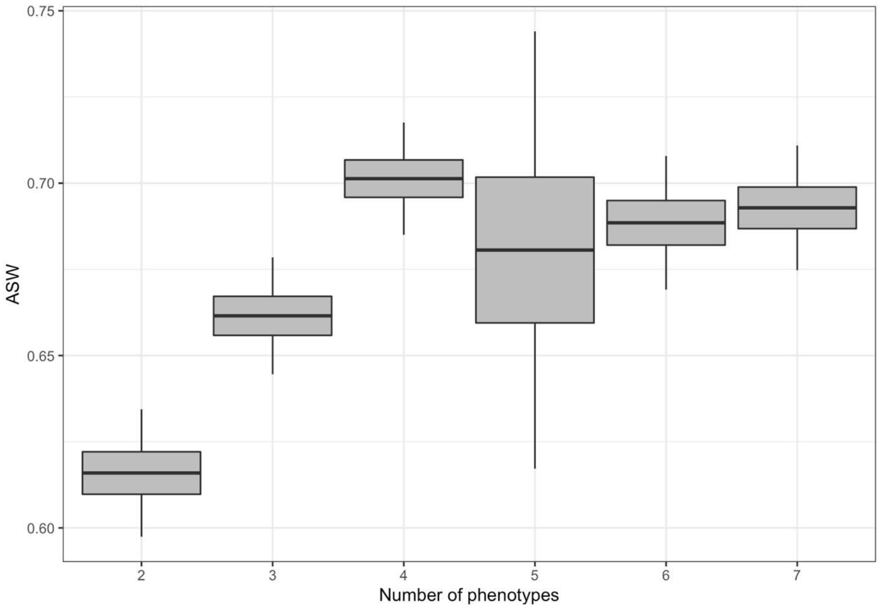

Figure S6: Profiles of wheeze phenotypes over time – Analysis of class stability with respect to changes in different sample sizes[1] of data

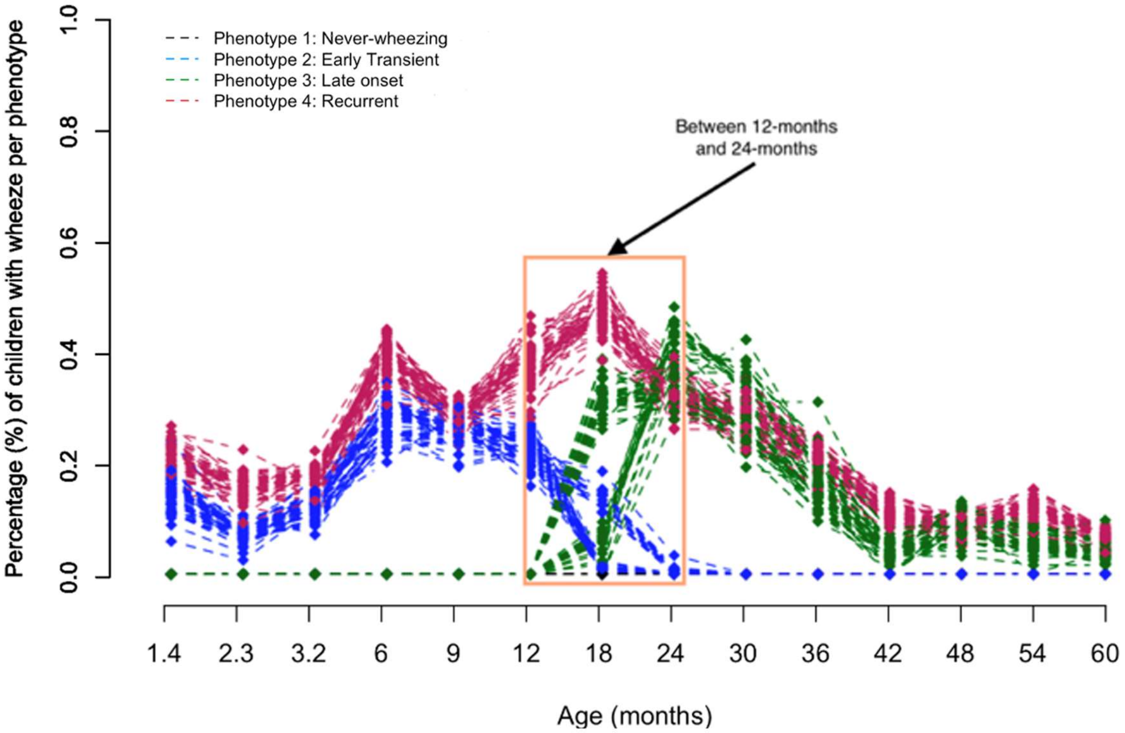

[1]We ran multiple iterations of the PAM algorithm while sampling random subsets of children of varying sample size with decrements of 10% from the full set of children until only half of the children were included. In each run, indicated by a separate line, 4 phenotypes was the optimal solution.

Table S7: BIC values for the LCA model in DCHS

Number of phenotypes	***BIC***
2	*6048.78*
3	*6092.89*
4	*6164.11*
5	*6238.73*
6	*6309.63*
7	*6381.43*

Figure S7: Characteristics of 4 wheeze phenotypes identified in the DCHS using LCA

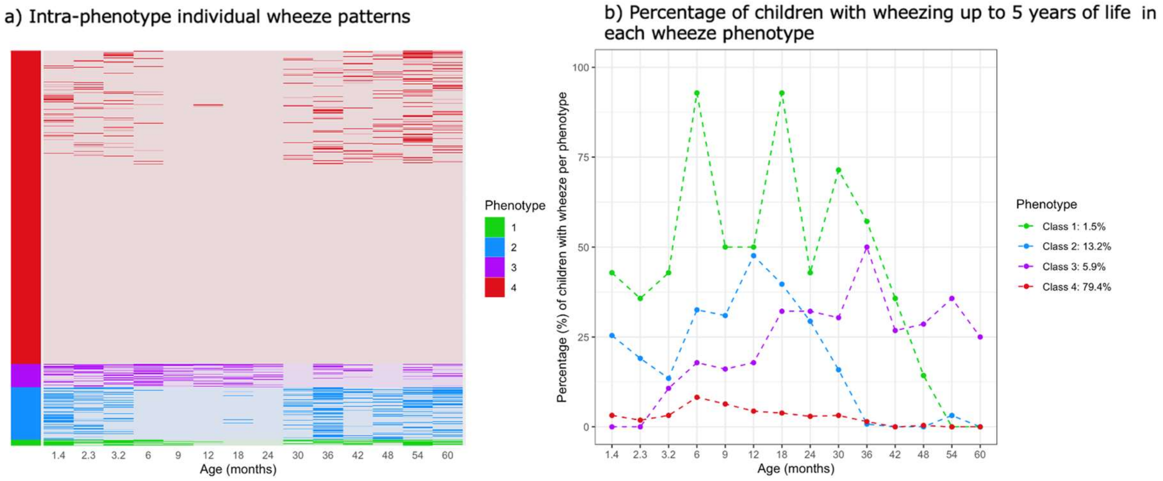

Figure S8: Point prevalence of current wheeze in ALSPAC by age

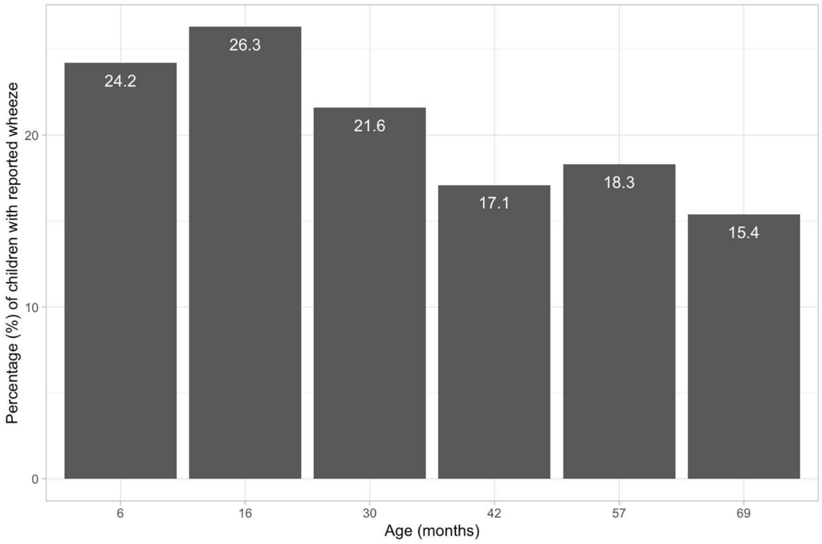

n=1635/6754 (6 months); n=1774/6754 (16 months); n=1459/6754 (30 months); n=1158/6754 (42 months); n=1233/6754 (57); months n=1038/6754 (69 months)

Figure S9: Plot of average silhouette width in ALSPAC to determine optimal number of clusters using PAM algorithm

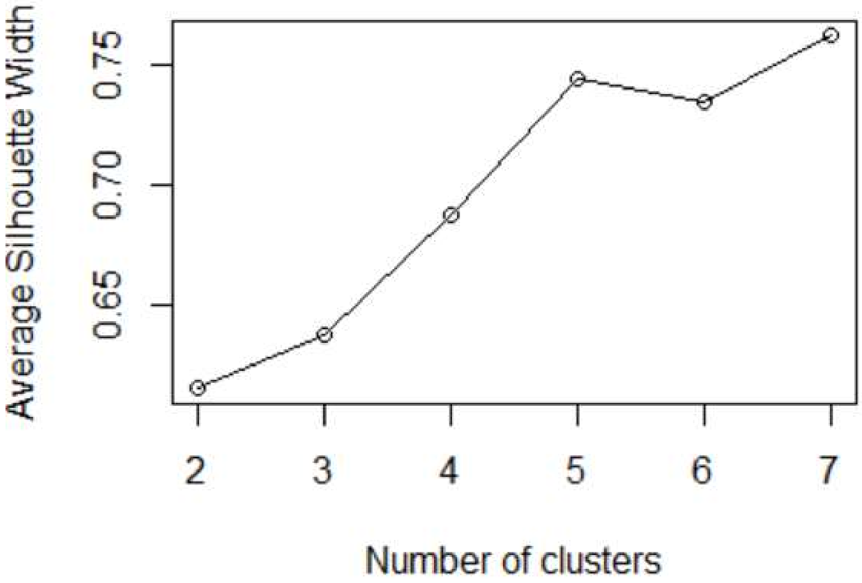

Figure S10: Characteristics of 5 wheeze phenotypes identified in ALSPAC

a) Percentage of children with reported wheeze in the first 5 years of life within each wheeze phenotype

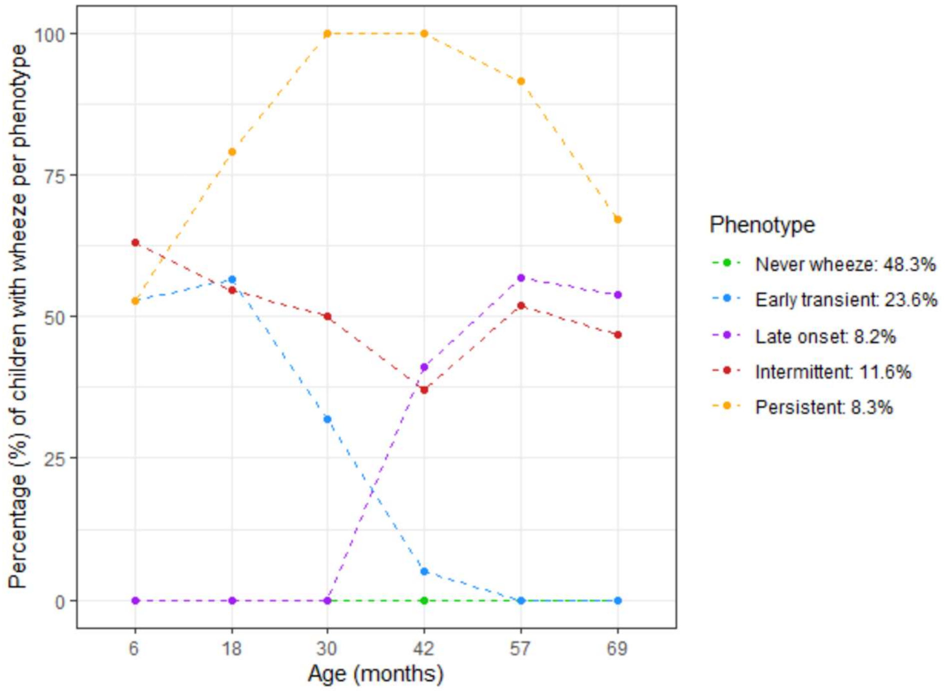

b) Intra-class individual wheezing patterns in ALSPAC

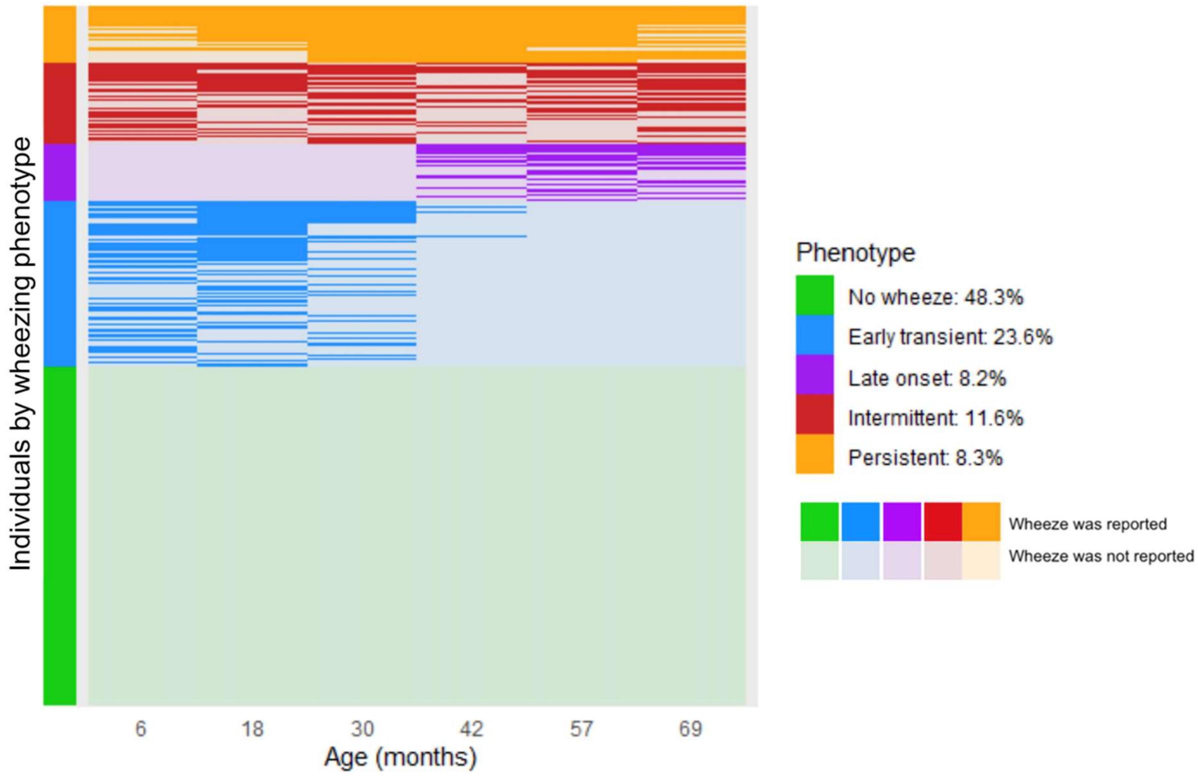

Comparison of ALSPAC phenotypes with LCA phenotypes

Henderson et al.[16] identified 6 wheeze phenotypes using data in the first 6 years of life in ALSPAC (Never/infrequent wheeze (59.3% of children), Transient early wheeze (16.3%), Prolonged early wheeze (8.9%), Intermediate onset wheeze (2.7%), Late onset wheeze (6.0%), and Persistent wheeze (6.9%)). There were notable differences compared with the current study, for example, we did not identify Prolonged early or Intermediate classes. No children in our Never wheeze phenotype wheezed in contrast to the sporadic wheezing evident in the LCA class. Consequently, PAM Never wheeze is smaller than that in the LCA study. In the LCA study, Late onset had >20% prevalence of wheeze up to 42 months; in the PAM model, no children wheezed before 42 months. The Early class was similar in both studies with regards to the timing of wheeze, with remission observed from 42 months onwards, however, in the LCA study, approximately 10% of children wheezed at 81 months; no children wheezed in the PAM model by 57 months.

Table S8: Unadjusted multinomial logistic regression of the association of early−life factors with wheezing phenotypes (reference class: Never wheezing)

	Unadjusted Effects					
**Phenotype Comparison**	**Phenotype 2: Early transient**		**Phenotype 3: Late onset**		**Phenotype 4: Recurrent**	
	**OR (95% CI)**	**p-value**	**OR (95% CI)**	**p-value**	**OR (95% CI)**	**p-value**
**LRTI**						
Number of LRTI episodes	2·59 (2·13, 3·15)	p < 0·0001	2·40 (1·91, 3·02)	p < 0·0001	4·06 (3·29, 5·02)	p < 0·0001
Hospitalized LRTI (vs ambulatory LRTI)	1·15 (0·67, 1·98)	p = 0·61	1·02 (0·50, 2·08)	P = 0·95	2·26 (1·31, 3·88)	p = 0·081
RSV-LRTI (vs RSV negative)	3·26 (1·80, 5·93)	p < 0·0001	2·12 (1·35, 4·61)	p < 0·029	4·10 (2·23, 7·55)	p < 0·0001
RV-LRTI (vs RV negative)	1·22 (0·69, 2·14)	p = 0·48	1·81 (0·93, 3·48)	p = 0·076	1·64 (0·91, 35·01)	p = 0·11
AV-LRTI (vs AV negative)	1·03 (0·54, 1·96)	p = 0·93	1·68 (0·82, 3·42)	p = 0·15	1·12 (0·57, 2·21)	p = 0·72
Influenza-LRTI (A or B or C) vs influenza negative	1·17 (0·52, 2·59)	p = 0·69	1·48 (0·61, 3·59)	p = 0·38	0·58 (0·23, 1·46)	p = 0·25
Parainfluenza-LRTI (1, 2, 3 or 4) vs parainfluenza negative	1·19 (0·52, 2·68)	p = 0·67	1·18 (0·46, 3·02)	p = 0·73	1·24 (0·54, 2·86)	p = 0·61
**Maternal Characteristics**						
Antenatal Smoking	1·22 (0·85, 1·74)	p = 0·28	1·53 (0·98, 2·39)	p = 0·059	1·75 (1·19, 2·56)	p = 0·0042
Postnatal Smoking	1·16 (0·82, 1·65)	p = 0·39	1·70 (1·09, 2·65)	p = 0·021	1·97 (1·33, 2·87)	p = 0·0012
Maternal Allergy	1·46 (0·73, 2·93)	p = 0·28	1·69 (0·73, 3·92)	p = 0·22	1·57 (0·74, 3·33)	p = 0·23
Antenatal Depression	0·72 (0·47, 1·10)	p = 0·13	0·89 (0·52, 1·41)	p = 0·65	1·18 (0·77, 1·80)	p = 0·46
Postnatal Depression	0·97 (0·66, 1·43)	p = 0·87	0·91 (0·54, 1·52)	p = 0·72	1·38 (0·92, 2·08)	p = 0·12
Antenatal Psychological Distress	1·22 (0·79, 1·87)	p = 0·36	1·03 (0·57, 1·84)	p = 0·91	1·93 (1·25, 2·99)	p = 0·0033
Postnatal Psychological Distress	1·08 (0·66, 1·77)	p = 0·76	1·18 (0·63, 2·22)	p = 0·611	2·01 (1·24, 3·26)	p = 0·0067
Antenatal IPV	0·73 (0·50, 1·06)	p = 0·11	0·87 (0·54, 1·40)	p = 0·56	1·35 (0·92, 1·99)	p = 0·11
Postnatal IPV	1·26 (0·89, 1·77)	p = 0·190	1·68 (1·08, 2·62)	p = 0·019	2·40 (1·64, 3·51)	p < 0·0001
Mode of delivery (Caesarean)	1·20 (0·81, 1·80)	p = 0·37	1·25 (0·76, 2·09)	p = 0·37	0·80 (0·49, 1·31)	p = 0·38
**Infant Characteristics**						
Sex (male vs female)	1·24 (0·90, 1·72)	p = 0·17	1·38 (0·90, 2·17)	p = 0·13	2·64 (1·79, 3·89)	p < 0·0001
Pre-term (<37 vs >=37 weeks)	0·90 (0·49, 1·31)	p = 0·65	1·21 (0·68, 2·13)	p = 0·51	1·55 (0·90, 2·35)	p = 0·059
HIV (exposed uninfected vs unexposed)	0·92 (0·63, 1·35)	p = 0·68	1·35 (0·79, 2·32)	p = 0·26	1·45 (0·91, 2·33)	p = 0·12
Exclusive Breast Feeding at 6 weeks	0·72 (0·51, 1·00)	p = 0·051	0·77 (0·50, 1·19)	p = 0·24	0·86 (0·59, 1·25)	p = 0·45

Season of Birth						
Autumn vs Summer	1·70 (1·09, 2·66)	p = 0·023	1·30 (0·72, 2·37)	p = 0·38	1·69 (1·02, 2·81)	p = 0·038
Winter vs Summer	0·84 (0·54, 1·32)	p = 0·46	0·66 (0·35, 1·22)	p = 0·18	0·80 (0·47, 1·34)	p = 0·39
Spring vs Summer	0·82 (0·51, 1·33)	p = 0·43	1·17 (0·65, 2·09)	p = 0·61	0·91 (0·53, 1·56)	p = 0·73
Antibiotic exposure	1·05 (0·61, 1·82)	p = 0·85	1·68 (0·80, 3·53)	p = 0·17	0·72 (0·43, 1·19)	p = 0·21
**Socio Economic Status**						
Maternal education						
Tertiary vs primary	1.40 (0.60, 3.25)	p = 0.42	1.76 (0.36, 8.51)	p = 0.48	0.47 (0.15, 1.46)	p = 0.19
Completed secondary vs primary	0.96 (0.50, 1.85)	P = 0.91	3.26 (0.95, 11.14)	p = 0.059	0.85 (0.42, 2.17)	P = 0.66
Secondary vs primary	1.04 (0.55, 1.94)	p = 0.89	2.66 (0.79, 8.93)	p = 0.11	0.83 (0.42, 1.61)	p = 0.58
Income						
R1 000 to R5 000 vs <R1 000	1.17 (0.83, 1.65)	p = 0.36	0.83 (0.53, 1.31)	p = 0.43	0.97 (0.65, 1.45)	p = 0.91
More than R5 000 vs <R1 000	0.93 (0.57, 1.72)	p = 0.98	0.81 (0.39, 1.67)	p = 0.58	1.41 (0.80, 2.47)	p = 0.23
Asset Ownership						
Low-Medium vs Low	1.21 (0.79, 1.85)	p = 0.35	1.90 (0.99, 3.61)	p = 0.051	1.10 (0.68, 1.79)	p = 0.68
Medium-High vs Low	0.78 (0.49, 1.25)	p = 0.31	1.70 (0.87, 3.32)	p = 0.12	0.76 (0.44, 1.30)	p = 0.31
High vs Low	1.17 (0.72, 1.90)	p = 0.51	2.77 (1.42, 5.54)	p = 0.0031	1.30 (0.76, 2.21)	p = 0.33
Household Size						
Small-Medium [4-5) vs Small [1-4)	0.89 (0.56, 1.43)	p = 0.65	1.39 (0.74, 2.58)	p = 0.29	1.12 (0.65, 1.92)	p = 0.67
Medium-Large [5-7) vs Small	0.79 (0.51, 1.20)	p = 0.27	1.06 (0.58, 1.91)	p = 0.84	1.19 (0.74, 1.92)	p = 0.45
Large vs Small [7-18]	1.12 (0.72, 1.75)	p = 0.59	1.79 (1.00, 3.21)	p = 0.051	1.21 (0.72, 2.04)	p = 0.46
**Lung function (at 6-weeks)**[1]						
R_{eE} (hPa.s.L^{-1})	0·99 (0·97, 1·02)	p = 0·65	1·02 (0·99, 1·04)	p = 0·13	1·02 (0·99, 1·04)	p = 0·081
X_{eE} (hPa.s.L^{-1})	0·99 (0·96, 1·03)	p = 0·76	0·98 (0·94, 1·02)	p = 0·31	0·94 (0·91, 0·97)	p = 0·0021

LRTI = Lower Respiratory Tract Infection; IPV = intimate partner violence, RSV = Respiratory Syncytial Virus; RV = Rhinoviruses, AV = adenovirus; R_{eE} = Respiratory resistance at the end of expiration; X_{eE} = Respiratory reactance at the end of expiration

[1]Lung function at 6-weeks was adjusted for height, sex, and ancestry.

Figure S1: Airway resistance at ages 6 weeks and 5 years in four wheeze clusters in DCHS (mean z-scores, 95% CI)

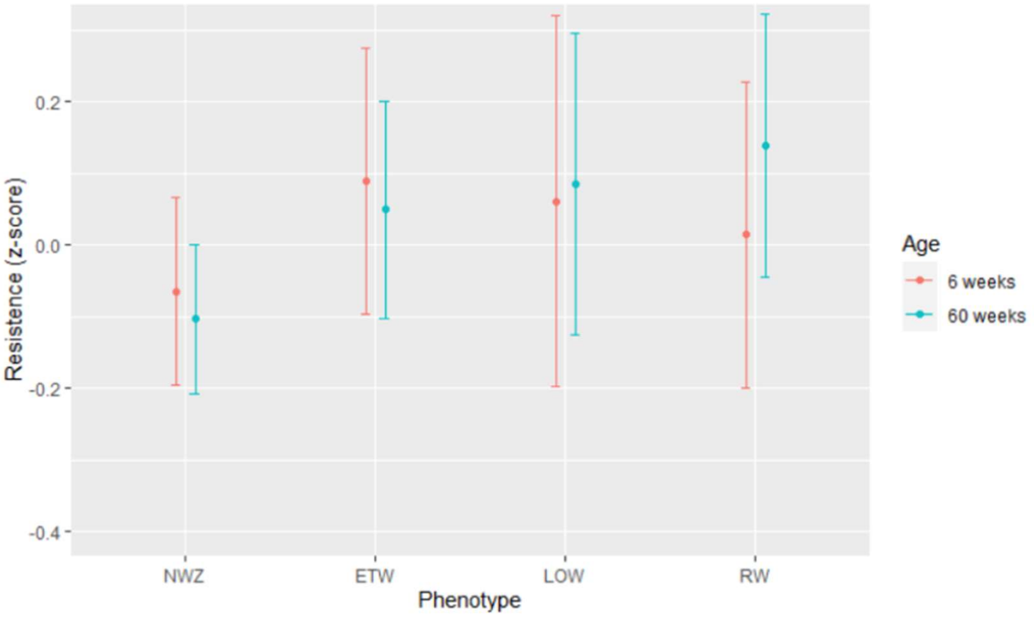

NWZ = Never wheeze; ETW = Early transient wheeze; LOW = Late onset wheeze; RW = Recurrent wheeze

Figure S2: Analysis of residuals plots assessing linearity of data (left), homogeneity of residuals variance (centre), and normality of residuals (right)

a) Respiratory resistance at the end of expiration (R_{eE})

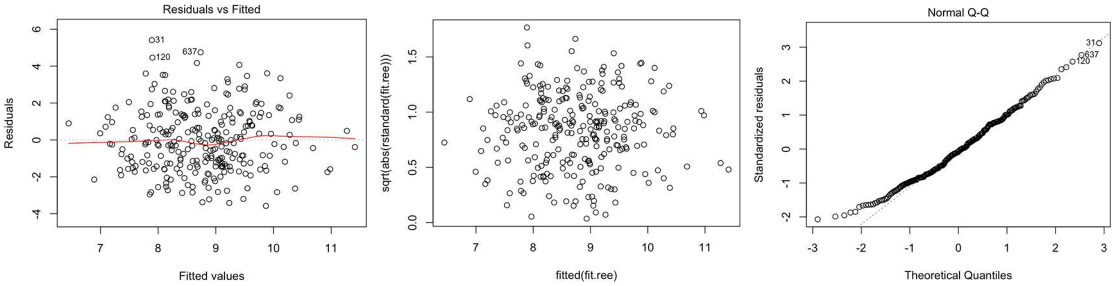

b) Respiratory reactance at the end of expiration (X_{eE})

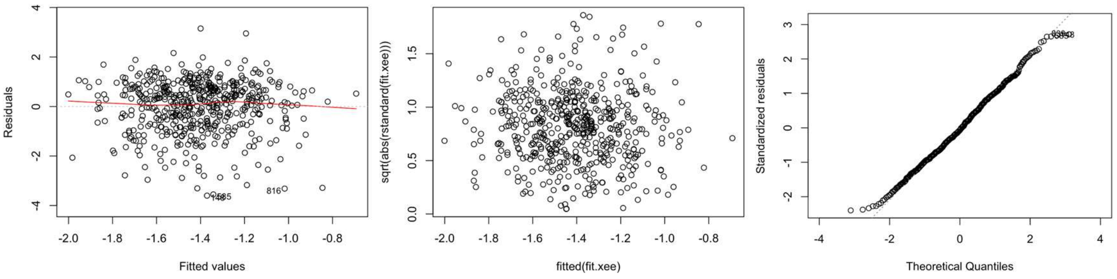

Figure S13: Average silhouette width to determine the optimal number of clusters using the PAM algorithm - application of PAM to binary wheezing outcomes

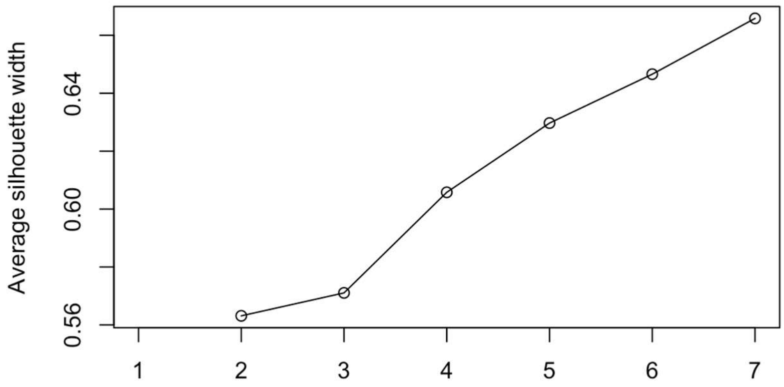

Figure S14: Characteristics of 4 wheeze phenotypes identified in DCHS - application of PAM to binary wheezing outcomes

a) Percentage of children with wheezing up to 5 years of life in each wheeze phenotype

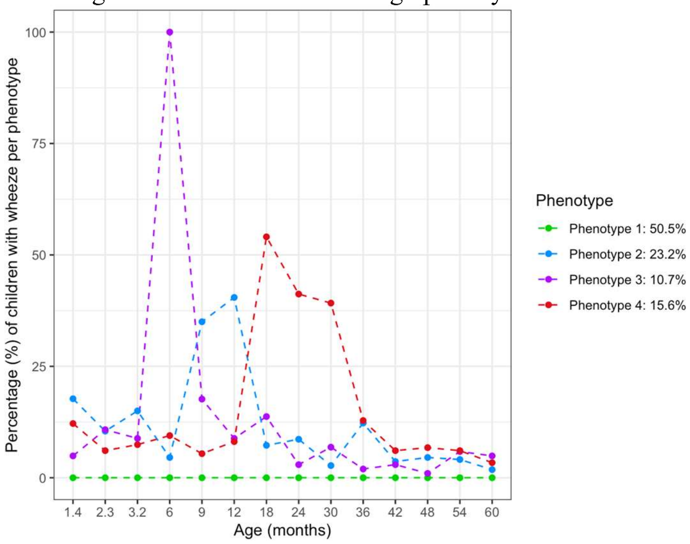

b) Intra-phenotype individual wheeze patterns

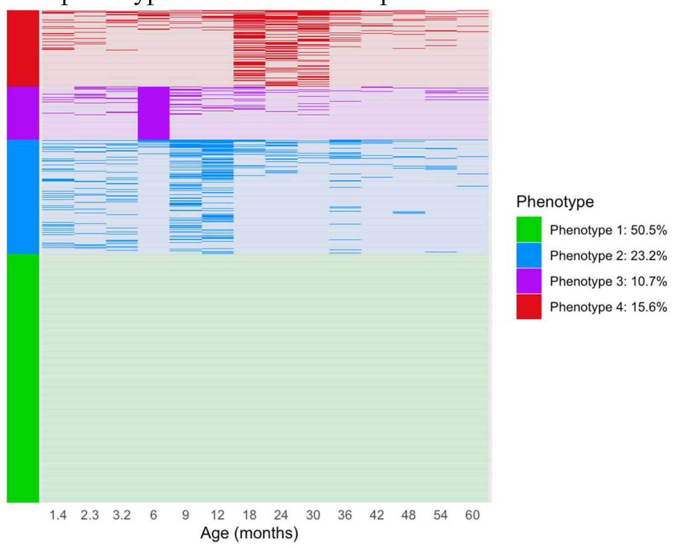

REFERENCES

1. Zar H, Barnett W, Myer L, Stein D, Nicol M. Investigating the early-life determinants of illness in Africa: the Drakenstein Child Health Study. Thorax 2015; **70**(6):592-4.

2. MacGinty RP, Lesosky M, Barnett W, Stein DJ, Zar HJ. Associations between maternal mental health and early child wheezing in a South African birth cohort. Pediatr Pulmonol 2018;**53**:741-54.

3. Zar HJ, Nduru P, Stadler JA, et al. Early-life respiratory syncytial virus lower respiratory tract infection in a South African birth cohort: epidemiology and effect on lung health. Lancet Glob Health 2020; **8**(10): e1316-e25.

4. Zar HJ, Barnett W, Stadler A, Gardner-Lubbe S, Myer L, Nicol MP. Aetiology of childhood pneumonia in a well vaccinated South African birth cohort: a nested case-control study of the Drakenstein Child Health Study. *Lancet Respir Med* 2016; **4**(6): 463-72

5. Gray D, Czövek D, Smith E, et al. Respiratory impedance in healthy unsedated South African infants: effects of maternal smoking. Respirology. 2015 Apr;20(3):467-73.

6. Gray DM, Czovek D, McMillan L, et al. Intra-breath measures of respiratory mechanics in healthy African infants detect risk of respiratory illness in early life. *Eur Respir J* 2019;**53**

7. Fraser A, Macdonald-Wallis C, Tilling K, et al. Cohort Profile: the Avon Longitudinal Study of Parents and Children: ALSPAC mothers cohort. Int J Epidemiol 2013;**42**:97-110.

8. Oksel C, Granell R, Haider S, et al. Distinguishing Wheezing Phenotypes from Infancy to Adolescence. A Pooled Analysis of Five Birth Cohorts. Ann Am Thorac Soc 2019;**16**:868-76.

9. Partitioning Around Medoids (Program PAM). Finding Groups in Data 1990. DOI: doi:10.1002/9780470316801.ch2

10. Kaufman LRPJ. Finding groups in data : an introduction to cluster analysis. New York: Wiley, 1990.

11. Arbelaitz, O., Gurrutxaga, I., Muguerza, J., Pérez, J. and Perona, I., 2013. An extensive comparative study of cluster validity indices. Pattern Recognition, **46**(1), 243-256.

12. Halkidi M, Batistakis Y, Vazirgiannis M. On clustering validation techniques. J Intell Inf Syst 2001;**17**(2-3):107-145.

13. Caliński T, Harabasz J. A dendrite method for cluster analysis. Communications in Statistics 1974;**3**(1):1-27.

14. Group, W., 2002. The Alcohol, Smoking and Substance Involvement Screening Test (ASSIST): development, reliability and feasibility. Addiction, **97**(9), 1183-1194.

15. Stein, D., Koen, N., Donald, K., et al. 2015. Investigating the psychosocial determinants of child health in Africa: The Drakenstein Child Health Study. Journal of Neuroscience Methods, **252**, 27-35.

16. Henderson J, Granell R, Heron J, et al. Associations of wheezing phenotypes in the first 6 years of life with atopy, lung function and airway responsiveness in mid-childhood. Thorax 2008;63:974-80.
